# The DNA damage response in advanced ovarian cancer: functional analysis combined with machine learning identifies signatures that correlate with chemotherapy sensitivity and patient outcome

**DOI:** 10.1038/s41416-023-02168-3

**Published:** 2023-02-21

**Authors:** Thomas D. J. Walker, Zahra F. Faraahi, Marcus J. Price, Amy Hawarden, Caitlin A. Waddell, Bryn Russell, Dominique M. Jones, Aiste McCormick, N. Gavrielides, S. Tyagi, Laura C. Woodhouse, Bethany Whalley, Connor Roberts, Emma J. Crosbie, Richard J. Edmondson

**Affiliations:** 1 Division of Cancer Sciences, Faculty of Biology, Medicine & Health, University of Manchester, M13 9WL Manchester, UK; 2 Early Phase Drug Development, Labcorp, Harrogate, HG3 UK; 3 Manchester University Hospitals NHS Foundation Trust, Oxford Road, Manchester, M13 9WL UK; 4 Department of Gynaecological Oncology, Glasgow Royal Infirmary, 16 Alexandra Parade, Glasgow, G31 2ER UK; 5 Christie NHS Foundation Trust, Manchester, UK; 6 Department of Obstetrics and Gynaecology, Manchester Academic Health Science Centre, Saint Mary’s Hospital, Oxford Road, Manchester, M13 9WL UK

**Keywords:** Ovarian cancer, Cancer therapeutic resistance

## Abstract

**Background:**

Ovarian cancers are hallmarked by chromosomal instability. New therapies deliver improved patient outcomes in relevant phenotypes, however therapy resistance and poor long-term survival signal requirements for better patient preselection. An impaired DNA damage response (DDR) is a major chemosensitivity determinant. Comprising five pathways, DDR redundancy is complex and rarely studied alongside chemoresistance influence from mitochondrial dysfunction. We developed functional assays to monitor DDR and mitochondrial states and trialled this suite on patient explants.

**Methods:**

We profiled DDR and mitochondrial signatures in cultures from 16 primary-setting ovarian cancer patients receiving platinum chemotherapy. Explant signature relationships to patient progression-free (PFS) and overall survival (OS) were assessed by multiple statistical and machine-learning methods.

**Results:**

DR dysregulation was wide-ranging. Defective HR (HRD) and NHEJ were near-mutually exclusive. HRD patients (44%) had increased SSB abrogation. HR competence was associated with perturbed mitochondria (78% vs 57% HRD) while every relapse patient harboured dysfunctional mitochondria. DDR signatures classified explant platinum cytotoxicity and mitochondrial dysregulation. Importantly, explant signatures classified patient PFS and OS.

**Conclusions:**

Whilst individual pathway scores are mechanistically insufficient to describe resistance, holistic DDR and mitochondrial states accurately predict patient survival. Our assay suite demonstrates promise for translational chemosensitivity prediction.

## Introduction

An impaired DNA damage response (DDR) is fundamental to the development of the genome instability that defines cancer [1]. The DDR is complex with pathway overlap but can be represented as five pathways; two for double-strand breaks (DSB) and three for single-strand breaks (SSB).

The role of homologous recombination (HR) pathway is the best-described DDR pathway. Tasked with the high-fidelity homology-based repair of DNA DSB, HR repair aligns to cell cycle to ensure DNA is repaired prior to mitosis or S phase [2, 3]. Non-homologous end joining (NHEJ) represents the second DSB DNA repair pathway in the cell. NHEJ can directly ligate broken DNA and can accommodate non-compatible sequences with non-complementary break overhangs (reviewed in ref. [4]). NHEJ operates throughout the cell cycle thus, whilst error-prone, is the predominant DSB repair DDR pathway in human cells.

Three DDR pathways each govern distinct forms of SSB DNA repair: The base-excision repair (BER) pathway responds to non-helix-distorting single-base lesions such as oxidised bases, deaminated bases, and alkylated bases that are caused by reactive oxygen species (ROS) or ionising radiation assault [5]. Nucleotide excision repair (NER) repairs helix-distorting bulky lesions and crosslinks [5, 6], while mismatch repair (MMR) responds to Watson-Crick mismatch base erroneous insertion, deletion, and mis-incorporations [7, 8].

However, the reality is that DDR pathway overlap and redundancy exists [9]. Moreover, the DDR needs to be seen in the context of other intracellular processes including mitochondrial (Mt) dysfunction and reactive oxygen species (ROS) both of which influence chemotherapy response [10]. Chemoresistance is multifaceted and driven by mechanistic and temporal factors connected to both the tumour microenvironment and the intrinsic ability of the ovarian cancer cell to resist chemotherapy (CT) [11]. Platinum CT is accepted to act by increasing intracellular ROS beyond a critical redox-homoeostasis threshold from which cancer cells cannot recover [10]. Chemoresistant cancer cells likely evolve rapid homoeostatic recovery or increased tolerances to accommodate the frequently-observed ROS abundance and oxidative stress increases [12] while perturbed ROS signalling enhances cell proliferation and survival [13]. As key producers and modulators of ROS [14], mitochondria are highly relevant to platinum CT efficacy. It is established that cancer cell Mt mutations act as oncogenomes [15] to influence oncogenesis [16] wherein chemoresistant cell Mt dysfunction associates with aggressive ovarian cancer subtypes, apoptotic resistance [17], and metastasis independent of the microenvironment [18].

Ovarian cancer is a heterogeneous set of diseases characterised by chromosomal instability and is an ideal model for profiling the DDR landscape [19]. The relevance of the DDR status to patient survival is exemplified by firstly the prognostic importance of *BRCA* gene mutation testing (as a means to presume HR functionality) currently operational in UK clinical practice, and secondly the success of PARP inhibitor therapy [20] to modulate patient tumour DDR response for increased progression-free longevity prior to patient relapse. In contrast to traditional cell line models, primary cultured patient cells offer a superior means to understand tumour heterogeneity (a) within a single tumour site, (b) across disparate sites within the same patient, and (c). between individual patients. Such facets permit deeper analytical integration of tumour DDR states for greater tumour diagnostic resolution. Although individual pathway aberrations have been described for HR [21, 22] and NHEJ [23, 24] in particular, to date a comprehensive assessment has not been carried out.

In order to understand how DDR deficiencies relate to ovarian cancer therapy response and patient survival, we developed a suite of ex vivo assays to test DDR function in live cells isolated from patients. This functional assay suite provides comprehensive DDR pathway metrics and incorporates mitochondrial dysfunction and ROS capacity scores to define the DDR landscape in ovarian cancer. The methods we report are robust and simple to run in a laboratory environment with low monetary and time expenses.

A preliminary explant “optimisation cohort” derived from 25 patient samples was profiled and corresponding patient outcome data were used to develop machine-learning models for patient survival. We subsequently refined and tested this approach on a separate explant “Validation cohort” derived from 29 patient samples. Furthermore, because CT elicits genomic assault, we ascertained whether neoadjuvant chemotherapy cycles prior to Interval Debulking Surgery (IDS) presented intrinsically disparate cancer explant DDR capacity profiles compared to explants from primary surgery (PS) patients. We determined that comprehensive live-cell assays integrated with patient outcome metrics can consistently successfully classify and predict patient progression-free and overall survival by multiple distinct machine-learning models.

## Materials and methods

### Ethics, recruitment and data collection

Samples were obtained from patients undergoing primary or delayed primary surgery for advanced ovarian cancer. Informed consent was obtained from all patients for sample and data collection and the study was approved by ethics (NHS National Research Ethics Service (NRES) Committee North West approval numbers 14/NW/1260 or 19/NW/0644 (collection date-dependent). Histological diagnosis was confirmed by specialist gynaecological pathologists. Patient data were obtained from patient records including time from diagnosis to first relapse (progression-free survival (PFS)) and time from diagnosis to death (overall survival (OS)).

### Explant establishment and routine culture

Solid tumour specimens and ascitic fluid samples were freshly transported. Acellular and calcified structures were excised from solid specimens prior to incubation with ≥0.1 U/ml Collagenase and 0.8 U/ml Dispase (Roche) and collection of resuspended solid tumour cells. Ascitic cultures were directly transferred into culture media.

Standard culture media comprised RPMI with 20% bovine serum (BS) 2 mM glutamine, 120 units/ml penicillin G sodium, 100 µg/ml streptomycin, and 0.5 µg/ml amphotericin B (all components were Gibco). Explants were maintained at 37 °C 5% CO_2_ and passaged at 70% confluency. Cryopreservation stocks were prepared from P0 in 90% BS 10% DMSO for all ex vivo cultures for prospective analysis. Successful explants were defined as those cultures which adhered to the following criteria: (1) a minimum of 90% epithelial cell density (by microscopy observation and characterisation, below); (2) amenable to a minimum of four passages prior to senescence; (3) exponential growth retention during usage across the entirety of the assay suite; (4) reached sufficient cell number required for the functional assay suite; (5) presented clear entry into senescence subsequent to functional assay completion.

### Explant characterisation

Cells were fixed in ice-cold methanol and exposed to 5% goat serum (Sigma, G9023) for 1 h at room temperature. Wells were treated with either anti-Ca125 (Abcam, ab1107), anti-Pax8 (Abcam, ab53490), anti-vimentin (Abcam, ab92547), or anti-Pan Cytokeratin (Merck, cbl234f) antibody (1:100 dilution in 5% Goat Serum) for 1 h at room temperature. Wells were washed and relevant primary antibodies, and unstained wells were incubated with Alexa Fluor 546 or 488 antibodies (Invitrogen, A-11003 and A-11008) at concentrations of 2 µg/ml for 1 h at room temperature. Cells were imaged with a Zeiss Axio Observer Z1 and Zen 2.3 software. Antibodies are provided in Supplementary Information Section 1.1. Mesenchymal cell type was monitored and <10% explant presence was deemed tolerable to avoid introducing bias into functional assay results. >=10% cultures were removed from analyses. Epithelial cells were not further isolated from cultures in order to retain relevance to the primary tumour source and avoid further artificial culling of the ex vivo phenotype proven important for HGSOC representation.

### Rad51 assay (HR)

HR was assessed as previously described [25]. Cycling cells were seeded at 40,000 cells/cm^2^ and incubated for 24 h. Cells were exposed to 200 mJ/cm^2^ of UV-C radiation, incubated for 2 h, and fixed in ice-cold methanol. Cells were permeabilised and treated with 1 µg/ml anti-phosphorylated H2AX (Millipore, 05-636-I) and 1:1000 anti-Rad51 (Abcam, ab133534) antibodies followed by exposure to 2 ug/ml Alexa Fluor 546 and 488 antibodies (Invitrogen, A-11003 and A-11008). Cells were imaged with a Zeiss Axio Observer Z1 and Zen 2.3 software. ImageJ software identified DAPI nuclear regions. A twofold increase in the average number of phosphorylated H2AX foci in irradiated versus control cells demonstrated sufficient cellular assault. Our previously validated threshold of a twofold increase in average Rad51 foci number versus control signalled a competent cell [25]. Antibodies are provided in Supplementary Information Section 1.2.

### In vitro cell extract assay (NHEJ)

The cell extract assay (NHEJ) used for the optimisation cohorts was carried out as previously described [23]. Further details are provided in Supplementary Information Section 1.3.

### Host-cell reactivation assay (NHEJ)

The Host-cell Reactivation system by Nagel et al. [26] was expanded to provide per-cell quantitative NHEJ pathway capacity monitoring of blunt end, 5’-3’ and 3’-5’ discontiguous dephosphorylated mismatched overhang DNA double-strand breaks. XL1-Blue supercompetent cells (200236; Agilent) were transformed with reconstituted pCMV6-AC-GFP plasmid (ps100010; Origene). EcoRI-HF (R3101S), SacI-HF (R3156S), KpnI-HF (R3142S), PmeI (R0560S), and Quick CIP (M0525) (New England Biolabs) were used to construct three dephosphorylated DSB plasmid conformations. Explants were plasmid transfected (lipofectamine LTX or 3000 (15338-100, 11668-027; Thermo Fisher)). Cultures were monitored at 24, 48 and 72 h. Fluorescence-governed NHEJ pathway activity was assessed qualitatively (microscopy) and quantitatively (flow cytometry). Event monitoring provides per-cell resolution of intra-explant NHEJ repair capacity variance and enables the detection of basal-level NHEJ activity. Ordinal plasmid condition scores were integrated to provide *Explant capacity scores* for explant NHEJ repair capacity. Further details are provided in Supplementary Information Sections 1.4 and 2.3.

### Single-cell gel electrophoresis assay (BER & NER)

The alkaline single-cell gel electrophoresis (comet) assay [27] was adopted to monitor BER and NER pathway capacities. The assays employ pathway-relevant DNA damaging mechanisms and pathway-specific inhibition agents. Pathways were monitored concurrently to minimise variation. BER and NER assays each comprised four incubation conditions containing 400,000 explant cells at 250,000 cells/ml. Antibiotics & antifungals were removed 24 h prior to assay preparation.

#### BER assault conditions

*Condition one*: Negative control (DMSO). *Condition two:* pathway repair blockade (10 µM Olaparib (A10111-100, Generon Ltd.)). *Condition three:* genomic assault (200 µM H_2_O_2_). *Condition four:* Damage during repair blockade (combined condition two and three agents). Cells were acclimatised for 60 min at 37 °C prior to treatment with Olaparib and H_2_O_2_ for 20 min on ice. Samples were washed with 4 °C dPBS, resuspended in 37 °C standard media, and incubated for 90 min at 37 °C 5% CO_2_.

#### NER assault conditions

*Condition one*: Negative control (DMSO). *Condition two:* pathway repair blockade (50 µg/ml aphidicolin (Tocris; CAS No: 38966-21-1)). *Condition three:* genomic assault (50 µM Benzo[a]pyrene (B[a]P) (Sigma)). *Condition four:* Damage during repair blockade (combined condition two and three agents). Cells were acclimatised for 30 min prior to treatment with aphidicolin. After 30 min, cells were treated with B[a]P and incubated for 150 min at 37 °C 5% CO_2_.

#### Routine comet assay conditions and score generation

Routine comet slide preparation, lysis, DNA unwinding, electrophoresis, imaging, and scoring were conducted as detailed in Supplementary Information Section 1.5. In all, ×10 magnification 172-megapixel images were scored using Trevigen Analysis Software (Trevigen). Between 6000 and 14,000 comets were routinely quantitated per each explant condition.

### Whole-exome sequencing (MMR)

In contrast to the other pathways which comprise multiple overlapping components, the MMR pathway can be sufficiently defined by a 22-gene panel without a need for functional analysis. SureSelect Human All Exon V6 (Agilent) libraries were prepared using explant DNA and samples were whole exome sequenced (WES) on a DNBSEQ™ NGS Platform (BGI Genomics, Hong Kong). Trimmed reads (Phred > = 34.4) were aligned to human reference build Hg19/GRCh37 using BWA [28] (99.954% average alignment) and variants called using SAMtools [29]. Variants were annotated by dbSNP [30], SnpEff [31], and Variant Effect Predictor [32]. Outputs were recalibrated and intersect and union cohorts generated. A 22-gene extended MMR panel [33] was extracted, and variant mutations were inspected by type, frequency, ORF location, and SnpEff & VEP ontological mutation Impact annotations. Additional WES and MMR pathway capacity score information is provided in Supplementary Information Section 1.6.

### Explant platinum cytotoxicity and GR_50_ modelling

Explant cells were seeded at 2000 cells per well in 96-well plates. Cells were PBS washed and triplicate wells incubated with a 12-point scale (0 µM, 1 µM, 4 µM, 8 µM, 16 µM, 32 µM, 64 µM, 128 µM, 256 µM, 512 µM, 1024 µM, 2048 µM) of carboplatin (A10182, Generon Ltd.) for 72 h alongside cell-free drug-media controls. Cells were supplemented with CellTiter 96® AQueous One reagent (Promega) and colorimetric absorbance was measured at 450 nm (FLUOstar Omega, BMG). Explant OD values were normalised against negative control values and intra- and inter-plate replicate variance was inspected. GR_50_ [34] growth-adjusted IC_50_ models were generated per explant. The GR_50_ approach was adopted to ensure resistant classification was de-coupled from any basal proliferation rates that are indistinguishable from resistant interpretation with traditional IC_50_ analysis. All explants were proliferating at the time of cytotoxicity analysis. A 48 µM threshold binomially classified explants as sensitive or resistant as detailed in Supplementary Information Section 1.7.

### Intra-explant reactive oxygen species (ROS) burden

The explant cell response to ROS assault was assayed by DCFDA (ab113851, Abcam). Cells were seeded at 25,000 cells per well and incubated with 20 µM DCFDA for 45 min at 37 °C 5% CO_2_ in the dark. Replicate wells were incubated with an 8-point scale (0 µM, 4 µM, 8 µM, 16 µM, 32 µM 64 µM, 128 µM, 256 µM) of Tert-Butyl Hydrogen Peroxide (TBHP). Fluorescence was measured at Ex/Em 485/535 nm (FLUOstar Omega, BMG) and scored as detailed in Supplementary Information Section 1.7.

### Intra-explant mitochondrial membrane function

Explant mitochondrial membrane potential was assayed by JC-10 (ab112134, Abcam) upon live-cell cultures. Cells were seeded at 25,000 cells per well and incubated with a 5-point scale (0 mM, 0.2 mM, 0.4 mM, 0.8 mM, 1.6 mM) of H_2_O_2_ for 75 min at 37 °C 5% CO_2_ in the dark. Wells were supplemented with 30 µM JC-10 and incubated for 60 min at 37 °C 5% CO_2_ in the dark. Fluorescence was measured at Ex/Em 490/525 nm and 540/590 nm (FLUOstar Omega, BMG) and scored as detailed in Supplementary Information Section 1.7.

### Statistical analysis, capacity scoring, classification, modelling, validation, dimension reduction

#### Software

*Operating systems*: Windows 7 & 10 × 64; Linux Ubuntu 14.04.6 LTS. Microsoft Excel (2016–2019) were used for clinical data collation. Microsoft Access (2016–2019+ were used for biobank-resolution (surgery-instance) databases. The R language [35] (3.4.3–4.0.5) and RStudio [36] (1.1.456–1.4.1106-5) were used for explant-resolution and patient-resolution databases. Over 30 packages were used in addition to *BaseR* (Supplementary Information Section 1.7).

#### Database handling

Assay suite data were pre-processed and contained within their own R environments. Explant-resolution summary data were mapped to biobank-resolution idents and hashed patient NHS idents to permit bidirectional data shuttling at explant, biobank, and patient levels. Where patient idents mapped to multiple explant tissues, explant-resolution bench-lab findings were merged to patient-resolution summaries by a framework that accommodated discordant values by multiple weightings (Supplementary Information Section 1.7).

#### Statistical analysis, classification, machine learning

Normality was assessed by Shapiro–Wilk or Kolmogorov–Smirnov tests and Q–Q plots. Heteroscedasticity was determined by Bartlett’s, Levene’s, or Fligner–Killeen’s. Parametric tests were *t* test, ANOVA, or Welch’s ANOVA with Turkey HSD, Dunnett’s test, Bonferroni correction, or Benjamini–Hochberg’s false discovery rate post hoc tests. Nonparametric tests included Wilcoxon rank-sum, Wilcoxon signed rank, or Kruskal Wallis tests with Dunn–Šidák multiple comparisons adjustment.

Discriminant analysis was employed for classification. Feed-forward perceptron artificial neural networks were conducted with 200 iterations & 50 repeats per network. Support vector machines used radial kernelized hyperplanes constructed from tune grids. Cross-validation used repeated Ƙ-folds, LOOCV and LGOCV/Monte Carlo methods. Imputed datasets were not required during the analysis.

## Results

We developed a series of functional DDR, cytotoxicity and metabolic assays and deployed these on patient tumour live-cell cultures. Multiple machine-learning approaches were explored to model and classify the relationship between holistic patient tumour DDR states and associated patient survival. First, we explored an initial “Optimisation cohort” which demonstrated preliminary patient survival classification success. We further expanded the functional assay suite and deployed this in a “Validation cohort” to obtain improved classification utility.

### Study pipeline and patient cohorts

The Optimisation cohort comprised 25 ovarian cancer patients recruited between 2011 and 2017. The Validation cohort comprised 29 ovarian cancer patients recruited between 2018 and 2020. High-grade serous ovarian cancer (HGSOC) subsets were extracted from both cohorts and termed HGS-Optimisation and HGS-Validation, respectively, to enable focused analyses (Supplementary Information Section 2.2).

PFS and OS follow-up data were available for 17 and 11 patients in the Optimisation and HGS-Optimisation cohorts whilst data was available for 16 and 13 patients for the Validation and HGS-Validation cohorts respectively. With the exception of performance status, no significant differences were observed between cohorts for disease characteristics, physiological parameters and treatment pathways (Supplementary Information Sections 2.1 and 2.4). All patients were treated with a combination of surgery and platinum-based chemotherapy. Tissue samples were taken at the time of surgery with 24/54 (44%) of patients having primary surgery whilst the remainder had surgery after three cycles of neoadjuvant chemotherapy.

### Optimisation cohort and preliminary findings

For the Optimisation cohort, 25 explants were established and the ex vivo assay suite was used to generate binomial *competent* or *defective* scores for HR, NHEJ, BER, NER and MMR pathway states. Traditional discriminant analysis used these DDR scores to classify overall survival of the 17 optimisation cohort patients with an AUC of 0.856 (Supplementary Information Section 2.5). When constrained to the HGS-Optimisation cohort, patient’s overall survival classification improved (AUC of 0.950), which was driven by a single misclassified sensitive patient. These findings illustrated the importance of profiling patient live tumour cells with functional DDR pathway capacity assays in order to classify patient survival.

### Validation cohort: a global DDR pathway capacity signature

In order to further explore and refine our DDR pathway capacity assays we developed a Validation cohort pipeline (Supplementary Information Section 2.2), whereby 38 explants were established from 29 patients (Supplementary Information Sections 2.1 and 2.6) of which 24 (62%) were HGSOC and the remaining 14 (38%) comprised other ovarian cancer histotypes (Supplementary Information Section 2.6A, B). Our explant establishment success rate was 88% with 22 patients having a single explant, five patients with two explants, and two patients with three explants (Supplementary Information Section 2.6C, D). Thus, in contrast to the Optimisation cohort, the Validation cohort allowed the exploration of tissue site heterogeneity. Details of the anatomical site of origin of explants, and growth characteristics are provided in Supplementary Information 2.6E, F. Growth rates did not associate with broad histotype, FIGO stage or site of disease.

**Fig. 1:**
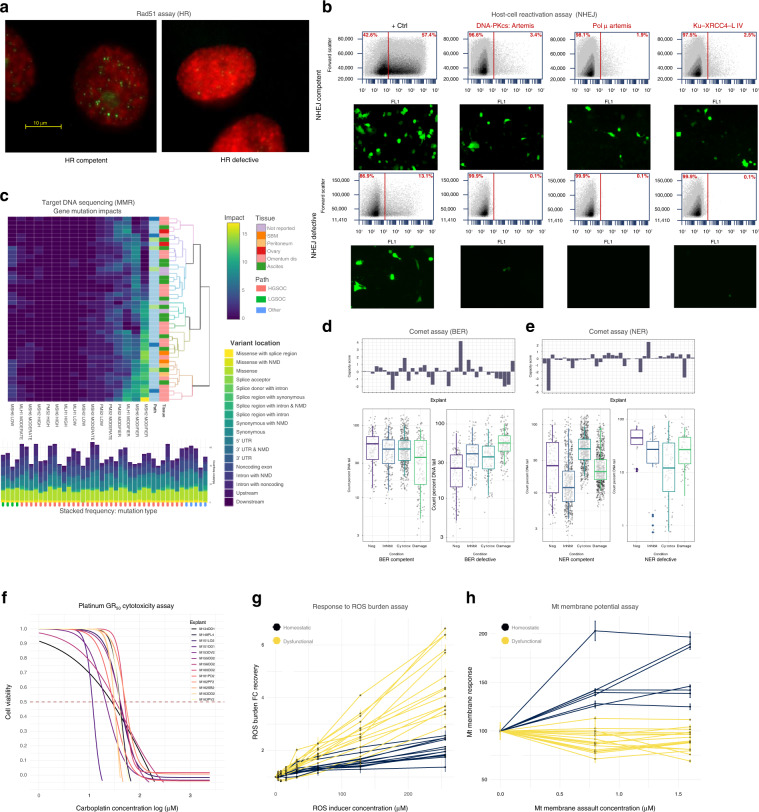
Representative explant-resolution DNA Damage Response (DDR) capacities in ovarian cancer. **a**–**e** Representative explant-resolution DNA damage response capacities in ovarian cancer. **f**–**h** Representative explant-resolution cytotoxic and metabolic tuning assays in ovarian cancer. **a** Rad51 assay (HR). Immunofluorescence-based explant cell scoring for Rad51 localisation and nucleoprotein filament formation following DNA DSB. The extent of repair was used to score explant HR pathway capacity. **b** Host-Cell Reactivation assay (NHEJ). Plasmid GFP expression cassettes were subject to three double-strand break conformation types and introduced into explant cells. Repaired plasmid double-strand breaks restores the cassette to permit GFP expression. The extent of repair was monitored qualitatively by fluorescence microscopy and quantitatively by flow cytometry. Each repaired conformation necessitates a particular cohort of NHEJ pathway components (maroon font). The extent of repair was used to score explant NHEJ pathway capacity. **c** Target DNA sequencing (MMR). Mutation status of MMR cohort genes was extracted from exome sequencing and assessed by mutation type, location, and frequency. VEP and SnpEff Impact Scores were used to score explant MMR pathway capacity. **d**, **e** Comet assays for BER and NER, respectively. Explants were subjected to BER or NER recipient single-strand break DNA damage in the presence or absence of pathway blockade. The extent of repair was measured by high-throughput comet assays (≈7000 comets typically per explant) and comet tail percent DNA metrics were used to score BER and NER pathway status. **f** Explant platinum cytotoxicity GR_50_ plots [34]. A range of cell viability is evident across explant cultures following treatment with increasing concentrations of carboplatin. **g** Explant response to reactive oxygen species (ROS) burden. Cells were treated with increasing concentrations of the ROS inducer TBHP. Cells were scored as homoeostatic or dysfunctional based on the DCFDA-derived extent of ROS retained in cells following a period of recovery. **h** Explant mitochondrial membrane potential status. Mitochondrial (Mt) membrane activity following Mt assault (percent-normalised to negative control cells). Homoeostatic explant mitochondrial membranes responded to assault c.f. dysfunctional explants whose mitochondrial membranes demonstrated no material response to membrane assault.

We profiled explant cultures using an extended functional DDR capacity assay panel which additionally included assays to determine explant platinum cytotoxicity state, explant response to ROS burden and explant mitochondrial membrane potential, Fig. 1. Assays showed no preferential bias between explant tissue types or broad tissue types (Supplementary Information Section 2.7A–G) thus demonstrating technical robustness.

### The DNA damage response pathway landscape in ovarian cancer explant cultures is varied

**Fig. 2:**
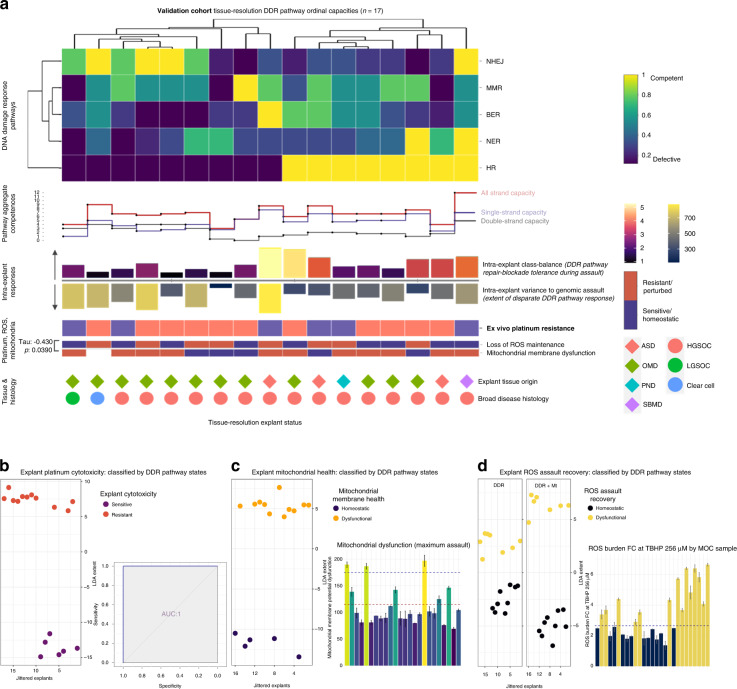
Validation cohort tissue-resolution explant DDR capacities with associated cytotoxicity and metabolic classifications. **a** DNA damage response (DDR) pathway states for Validation cohort explants. Each column is an explant and each row is a DDR pathway capacity. A range of defective pathway signatures are evident. Clustering resolves Host-Cell Reactivation (NHEJ) and Rad51 (HR) assays to almost diametrically opposed DSB repair capacity states. Aggregate pathway competences sum double-strand break, single-strand break, and all strand break capacities. Intra-explant class-balance bar plots depict extent-of-cell-viability survivorship following repair blockade, and during genomic assault. **b** Explant platinum cytotoxicity was completely classified by the five DDR pathway capacity states (discriminant analysis ROC AUC of 1). **c** The five DDR pathway capacity states completely classify mitochondrial membrane health (discriminant analysis ROC AUC of 1). **d** Explant response to ROS assaults was described by the five DDR pathway capacity states (AUC of 1) with distinct but limited discrimination distances (left plot). A marked increased discriminatory distance was obtained when supplemented with mitochondrial membrane status (right plot). An explant mitochondrial membrane state and its ability to recover a ROS burden were inversely correlated (*P* = 0.0390).

Each explant demonstrated at least one dysfunctional DSB pathway and one dysfunctional SSB pathway. HR-defective status was associated with a greater dysfunction in the remaining pathways such that at least three DDR pathways per explant culture were highly perturbed when HR pathways are defective (Fig. 2a).

The combination of (1) known starting cell numbers during (2) per-cell quantitation assessment across assay conditions permits a “class-balance” fold change calculation. This monitors for presence of hyper-resistant phenotype cell subpopulations which could otherwise be masked below the noise threshold of the explant global average capacity. The extent-of-cell-viability survivorship following repair blockade during genomic assault was examined using intra-explant class balance, Fig. 2a. A wide range of survivorship was evident across the explants. HR-competent explants demonstrated increased tolerance to repair blockade during assault with higher overall cell survivorship in contrast to HR-defective explants which contained overall reduced cell survivorship bias. These observations mechanistically agree with HR competence states and associated tumour resistance.

Next, the intra-explant cell population extent-of-variance following genomic assault was determined to be wide-ranging, Fig. 2a. HR-competent explants exhibited decreased variance in their response to genomic assault in contrast to HR-defective explants which overall demonstrated increased per-cell variance range to steady genomic assault. This greater observed intra-explant inter-cell heterogeneity is concordant with the greater loss of DDR pathway capacities in HR-defective cells and is in contrast to observed reduced variance in HR-competent cells as a consequence of tighter rebuttal to aggregate genomic instability during tumour evolution.

In all, 11 out of 17 (65%) explant cultures were platinum-resistant (Gr_50_ > = 48) with all explants actively proliferating for the entirety of the assay. This was equally split across both HR-competent and HR-defective explants, indicative that platinum relation to the DDR landscape is complex and is not a direct response to any individual DDR pathway state.

The explant response to ROS burden was wide-ranging across the DDR pathway explant signatures. Five out of eight (62.5%) HR-competent and four out of nine (44%) HR-defective explants were homoeostatic for ROS burden control (Fig. 2a).

Mitochondrial membrane potential signatures were largely dysfunctional across the explant cohort. Four out of five (80%) of platinum-resistant HR-competent and two out of three (66%) platinum-sensitive HR-competent explants exhibited perturbed mitochondrial membrane potentials. Three out of six (50%) platinum-resistant HR-defective explants and three out of three (100%) platinum-sensitive HR-defective explants exhibited perturbed mitochondrial membrane potentials indicative of no direct platinum associations. Mitochondrial membrane dysfunction and ROS burden control were neither co-dependent with nor correlated with any of the five DDR capacities or platinum cytotoxicity states (Supplementary Information Section 2.8C).

We observed a modest significant inverse correlation between explant mitochondrial membrane health and their ability to recover from a ROS assault (Kendall’s tau: −0.430, *P* = 0.0390). One out of 17 (5.9%) explants was homoeostatic for both ROS and mitochondrial membrane function, while three out of 17 (17.6%) explants were perturbed for both ROS and mitochondrial membrane function. In all, 13 out of 17 (76%) displayed inverse states (Fig. 2a).

### The ovarian cancer explant DDR pathway landscape classifies ex vivo platinum cytotoxicity and metabolic activity states

We next investigated whether explant platinum cytotoxicity states could be predicted by the observed DDR pathway capacity signature. Using discriminant analysis, we discovered that explant platinum cytotoxicity was completely classified by capacity states of the five DDR pathways profiled by our ex vivo assays (AUC: 1) (Fig. 2b). The greatest DDR pathway-derived discriminant coefficients (Supplementary Information Section 2.9A) were NHEJ status (11.42) and MMR status (4.34) indicative of the importance of both DSB and SSB DDR pathway status in cell tolerance of platinum agents. The greatest non-DDR-capacity discriminant coefficient was intra-explant extent-of-cell-viability survivorship (13.31) which mirrors an aggressive cell-viability phenotype and supports the relevance of survivorship bias metrics within explant analysis.

We discovered that complete discrimination of mitochondrial membrane health was achieved by the five DDR pathway capacity states (AUC of 1) (Fig. 2c). The discriminatory distance was driven by HR (17.44) and NHEJ (15.71) DDR pathway-derived coefficients and by intra-explant extent-of-cell-viability survivorship (e.g., 25.28) (Supplementary Information Section 2.9B).

An explant’s ability to respond to a ROS assault was described by the five DDR pathway capacity states (AUC of 1) with a distinct and limited discrimination distance (Fig. 2d). Given (i) our observed inverse correlation with explant mitochondrial dysfunction (above), and (ii) the mechanistic link between mitochondrial activity-derived ROS generation and a cell’s efficacy to control cytoplasmic ROS levels, we supplemented the five DDR pathway capacity signatures with the known mitochondrial membrane status and obtained a markedly greater discriminatory distance driven by a negative mitochondrial membrane dysfunction coefficient (−9.38) (Supplementary Information Section 2.9C).

### Patient summary analysis of explant DDR pathway, cytotoxicity and metabolic states

Next, we examined patient-level data by developing a patient score which accommodated the presence of multiple explants per patient. An algorithm ensured consistent summarising and was weighted by DDR capacity values, range of intra-explant variances, inter-explant heterogeneity, and the degree of class-balance cell survivorship (Supplementary Information Section 1.7).

**Fig. 3:**
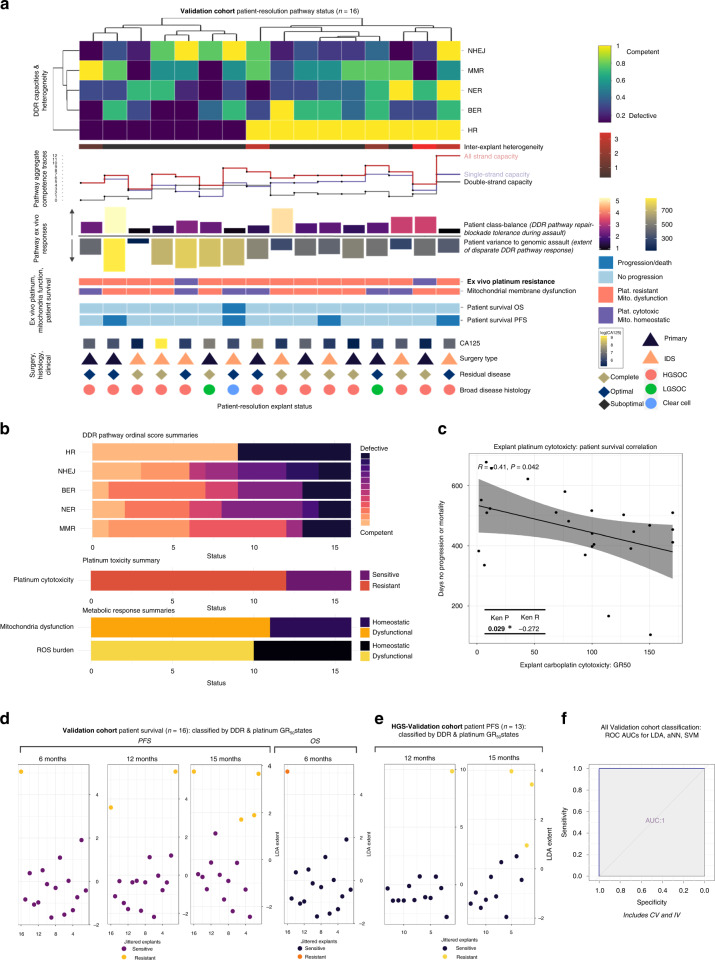
Validation cohort patient-resolution explant DDR capacities with platinum survival correlation and patient classifications. **a** DNA damage response landscape in relation to patient survival. Each column is an Validation cohort patient and each row is a DNA damage response pathway capacity. Aggregate pathway competences sum double-strand break, single-strand break, and all strand break capacities. Intra-explant class-balance bar plots depict extent-of-cell-viability survivorship following repair blockade, and during genomic assault. **b** Summary bar plots of frequency of DDR pathway capacity metrics, platinum cytotoxicity, mitochondrial dysfunction, and response to ROS burden. **c** A modest inverse correlation between explant platinum cytotoxicity and patient progression-free or overall survival was observed (*r* = 00.41, Pearson *P* = 0.042; Kendall’s tau *P* = 0.029). **d** DDR pathway capacity signatures and platinum cytotoxicity states can completely classify Validation cohort patient progression-free survival and overall survival status by discriminant analysis at 6, 12 and 15 months (ROC AUC of 1—see (**f**)). **e** DDR pathway capacity signatures and platinum cytotoxicity states can completely classify HGS-Validation cohort patient progression-free survival by discriminant analysis at 12 months and 15 months (ROC AUC of 1—see (**f**)). **f** Complete classifications were maintained for Validation and HGS-Validation cohorts up to 15-month progression-free survival with the use of non-linear feed-forward multilayer perceptron artificial neural networks (aNN) or radial basis function (RBF) kernel support vector machines (SVM) in conjunction with cross-validation and internal validation (ROC AUC of 1). Validation cohort 6-month PFS and 6-month OS, and HGS-Validation cohort 12-month PFS each contain a single resistant patient and thus were unsuitable for data partitioned internal validation.

Dendrogrammatic clustering of patients by DDR capacity states resolves the two DSB pathways to be diametrically opposed (Fig. 3a), with at least 1 DSB and 1 SSB defective pathway for each patient. HR-defective status associated with greater dysfunction in remaining pathways such that at least three DDR pathways per patient are highly perturbed when HR pathways are defective. No significant difference was observed between the extent of patient DDR pathway competencies and patient IDS or PS treatment routes (*P* value ranges: 0.308–0.874; Supplementary Information Section 2.10) indicative that stand-break repair competencies profiled here were tumour-evolution derived events rather than IDS CT-derived clinical occurrences. No patient had complete dysfunction across the entire DDR pathway complement.

The inter-explant heterogeneity score denotes that four out of nine (44.4%) HR-competent patients demonstrated inter-explant heterogeneity in contrast to one out of seven (14.2%) HR-defective patients, Fig. 3a. The inter-explant heterogeneity score did not directly associate with patient PFS or OS.

Wide ranges of repair-blockade tolerances during genomic assault and intra-explant cell variances were retained across the patient cohort. Patients presented similar trends to explant cultures wherein HR-competent patients had overall increased viability tolerances in contrast to HR-defective patients. HR-competent patients presented decreased per-cell variance ranges in response to the genomic assault, in contrast to HR-defective patients (Fig. 3a).

Follow-up confirmed that 12 out of 16 (75%) patients were platinum-resistant (relapsed with a treatment-free interval of less than 182 days) and three out of four (75%) patients who progressed were platinum cytotoxicity resistant. Seven out of nine (77.8%) HR-competent and five out of seven (71.4%) HR-defective patients were platinum cytotoxicity resistant which consolidated the explant-resolution observation that platinum relation to the DDR landscape is complex and not a direct response to any individual DDR pathway capacity.

Mitochondrial membrane functionality was perturbed in seven of nine (78%) HR-competent patients but only four of seven (57%) HR-defective patients. Every patient who relapsed or died exhibited perturbed mitochondrial membrane functionality (Fig. 3a).

To summarise these findings, DDR pathway dysfunction was wide-ranging across our patient cohort. Seven out of 16 (44%) patients were HR pathway defective while a range of NHEJ, BER, NER and MMR competence scores were evident. Most patients presented a range of partially perturbed or basal competence signatures. The majority of the patient cohort presented aberrant ROS control and dysfunctional mitochondrial membrane potentials (Fig. 3b).

We next assessed whether explant platinum cytotoxicity assays offer direct correlation utility with patient survival. A modest inverse correlation exists between explant platinum cytotoxicity and progression-free or overall survival (*r* = −0.41, Pearson *P* = 0.042; Kendall tau = −0.272, Kendall *P* = 0.029, Fig. 3c).

### Ovarian cancer patient PFS and OS can be classified by the DDR landscape and platinum cytotoxicity states

We sought to determine whether patient-summarised DNA damage response pathway capacity signatures and associated platinum cytotoxicity could be used to classify patient progression-free survival or overall survival. Figure 3d depicts patient survival classification for the Validation cohort by summarised explant DDR capacity signatures and platinum cytotoxicity at 6 months, 12 months and 15 months (maximum available follow-up duration at this time). One patient progressed within 6 months, two patients progressed within 12 months, and four patients progressed within 15 months. A single Validation cohort patient died within 6 months during this 15-month period. DDR pathway capacity signatures and platinum cytotoxicity states can completely classify Validation cohort patient progression-free survival and overall survival status by discriminant analysis (AUC of 1; Fig. 3f). This observation remained true when limited to patients with HGSOC pathology. Figure 3e depicts HGSOC patient survival classification by summarised explant DDR capacity signatures and platinum cytotoxicity at 12 months and 15 months. No HGS-Validation cohort patients progressed within 6 months, one patient progressed within 12 months, and three patients progressed within 15 months. No HGS-Validation cohort patients died within this 15-month period. DDR pathway capacity signatures and platinum cytotoxicity state completely classified HGS-Validation cohort progression-free survival by discriminant analysis (AUC of 1; Fig. 3f).

To assess whether the observed classification efficacy was limited to an individual approach, we adopted additional non-linear machine-learning approaches that included perceptron artificial neural networks (aNN) and radial basis function (RBF) kernel support vector machines (SVM) in conjunction with cross-validation and internal validation. Complete classifications were achieved for Validation and HGS-Validation cohorts up to 15-month progression-free survival by aNN and RBF-SVM approaches (AUC of 1; Fig. 3f).

### Dimension reduction identifies DDR drivers of PFS

**Fig. 4:**
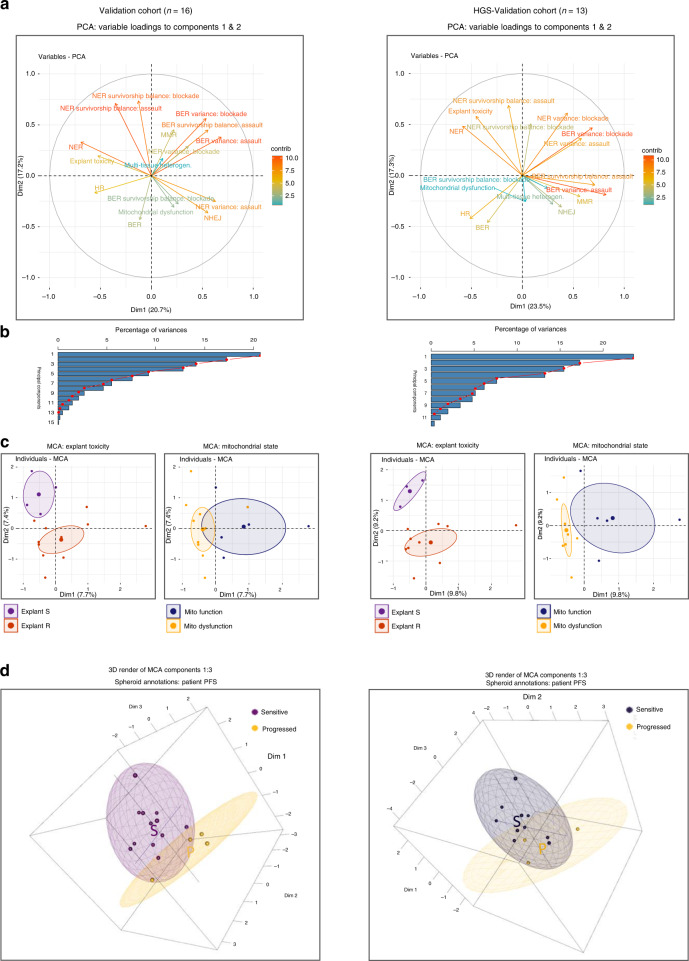
DDR capacity contributions to patient classification: dimension reduction. Left column: Validation cohort. Right column: HGS-Validation cohort. **a** Principal component analysis (PCA) dimension reduction variable loading compass plots of DDR pathway capacity functional assay panel to patient resistance, coloured by their contributions to components one and two. HR, NHEJ, NER and MMR capacity states occupy distinct directionally within both cohorts and contribute the most. Non-DDR capacity metrics (intra-explant extent-of-variance, survivorship class balance, and explant platinum cytotoxicity) also strongly contribute. **b** Associated PCA variance scree plots. **c** Multiple correspondence analysis (MCA) dimension reduction of platinum cytotoxicity and mitochondrial membrane dysfunction states classified by DDR pathway capacities. Platinum cytotoxicity ellipses (left insets) present clear separation in the Validation and HGS-Validation cohorts. mitochondrial membrane dysfunction ellipses (right insets) present distinct but overlapping regions in the Validation cohort; and fully partitioned ellipses in the HGS-Validation cohort. **d** Patient PFS classification by explant DDR and platinum status constructed from the first three MCA components and plotted into three-dimensional space. Spheroids provide patient survival classification group clusters. Distinct spheroids are visible for Validation and HGS-Validation cohort patient survival classes.

Principal component analysis (PCA) dimension reduction identified that HR, NHEJ, NER and MMR capacities contribute to patient resistance classification, Fig. 4. Non-DDR capacity metrics such as intra-explant extent-of-variance, survivorship class balance, and explant platinum cytotoxicity also strongly contributed.

Finally, multiple correspondence analysis (MCA) dimension reduction was used to provide an unsupervised view of the data. MCA identified separating clusters corresponding to explant cytotoxicity and mitochondrial status (Fig. 4c) and progression-free survival (Fig. 4d), thus confirming the ability of DDR signatures to predict ex vivo and clinical outcomes.

## Discussion

We present here a suite of functional assays to describe the status of the five canonical DDR pathways. Combined with ex vivo platinum cytotoxicity and mitochondrial membrane health, these functional DDR capacity assays were able to classify ovarian cancer patients progression-free and overall survival in our Validation cohort analysis. These assays are rapid, low-cost, and simple to implement in a laboratory environment and so have the potential as a possible triage service in the translational setting.

Chemosensitivity is a multifaceted phenomenon yet the intrinsic ability of a cell to repair DNA in response to chemotherapy-induced damage is a fundamental factor. Indeed, at least some of the recognised mechanisms of drug resistance, including drug efflux, may associate with an impaired DNA damage response [37]. Although extensively described, the role of homologous recombination has generally been studied in isolation and the role of alternative pathways are often overlooked. Here, we attempted to employ a range of assays which reflect a holistic picture of the DDR at an explant and patient level.

For a disease hallmarked by extensive chromosome instability it is perhaps not surprising that discrete signature patterns of DDR dysregulation were not seen across the cohorts of high-grade serous cancers studied here. Instead, we observed almost all permutations of DDR dysregulation with a preponderance towards more pathway dysregulation in HRD tumours than the HRC cohort. Our finding that the two double-strand repair pathways show near mutual exclusivity is interesting and suggests that strategies to identify druggable targets in the NHEJ pathway would have clinical utility in complementing PARP inhibitors, which preferentially target HRD tumours.

We also included ovarian tumours with a non-high-grade serous morphology. These tumours have different molecular drivers including kras/braf and PTEN [38–40]. The similar degree of DDR dysregulation that these tumours display to HGSOC encourages the notion a DDR signature may be a useful overlay to morphology in a wide variety of solid tumours.

All tumours used in this study were collected during primary treatment and all patients received carboplatin combined with paclitaxel as the mainstay of their chemotherapy. No statistically significant differences in DDR capacity states were observed between PS and IDS patients, indicative that observed DDR capacity failures reflect inherent ovarian tumour cell evolution rather than CT-induced clinical consequences. At the time of study recruitment PARP inhibitor therapy was not available as maintenance therapy with no patient receiving this before first relapse. Our ability to classify and predict outcomes for this cohort, therefore, represents a model for predicting response to platinum/taxane combination therapy only. Nevertheless, this model could now be studied as a potential tool to predict chemosensitivity to these agents, both of which are used extensively in the relapse setting. Furthermore, the subset of HRD ovarian cancer patients who are eligible for PARPi therapy often benefit from a significant initial response which unfortunately can lead to long-term acquired resistance wherein the extent of response corresponds to resistance severity (reviewed in ref. [41]).

The models generated here compare favourably with other methods of classification that have been proposed for high-grade serous cancer including DNA [22, 42] and RNA [43] based models. Moreover, the functional approach to these assays is justified by the incremental improvement in prediction seen with our five DDR assays compared to recent studies using an IHC approach to monitor three DDR pathways [44], and similar results for three DDR pathways in glioblastoma multiforme [45] with a panel including the host-cell reactivation system used here [26].

It is unsurprising that all five DDR pathways are required to generate an accurate predictive signature. Although the association of HR repair status with chemoresistance is known, an emphasis on any single pathway is mechanistically insufficient to capture compensatory and reciprocal activity across the remaining DDR pathways [5]. On the one hand XRCC1 actuation within the predominant SSB repair PARP-dependent BER pathway associates with poor clinical outcomes, whilst XRCC1-deficient [46] or APE1/Ref-1-silenced cells [47] are sensitised to cisplatin. Similarly, loss [48] or alternative-splicing [49] of the NER pathway component ERCC1 or loss of XPF [50] associates with platinum sensitivity in cells. These SSB pathway reports appear to align with the HR pathway notion that functional repair associates with poorer outcomes. Conversely, MMR has been reported to both offer [8] and not offer [51] prognostic significance, whilst NHEJ defectiveness can associate with sensitive and resistant phenotypes which can occur through 53BP1-derived NHEJ loss to reactivate HR competence via a *BRCA*-independent manner [52].

Mitochondria are central to a host of fundamental physiological processes and contribute to ROS generation and control [10]. Increased ROS abundance and oxidative stress are frequent events in ovarian cancer, and chemotherapy elevates ROS levels to alter cancer cell redox-homoeostasis [12]. We observed that seven of nine (78%) HR-competent patients harboured dysfunctional mitochondrial membranes in contrast to four of seven (57%) of HR-defective patients. Within the platinum cytotoxicity assay resistant subset, this partition further increased wherein six out of seven (86%) of HR-competent patients were mitochondrial membrane dysfunctional vs two out of five (40%) in HR-defective patients, and every relapse or mortality event contained perturbed mitochondria functionality. Furthermore, membrane action and ROS assault recovery were significantly inversely correlated. Of interest, ovarian cancer ex vivo mitochondrial membrane health can be fully classified by the DDR landscape signature which was driven predominantly by HR, NHEJ, MMR, and intra-tumour class balance. This was surprising but could be explained by the possibility that, in the presence of a DDR-competent phenotype, a membrane-perturbed oncogenic mitochondrion will drive a loss of ROS homoeostasis and thus evolutionarily enrich for ROS tolerances beyond the necessary chemo-induced cytotoxic threshold and promote chemoresistance.

Together, our findings show that the complexity and heterogeneity of the DDR response in cancer can be disentangled using a suite of well-defined assays representing both the five major pathways and more global representations of the DDR. The resulting signatures appear to have at least an association with the patient outcome although whether they have clinical utility to assess the DDR as a predictive tool to aid patient management requires further study, particularly given the multiple factors that may be responsible for determining chemotherapy resistance. Future study should focus on critical clinical decision points, such as for relapsed disease where the decision to treat is not always obvious. Although we have limited this study to ovarian cancer, the inclusion of multiple subtypes with different molecular drivers, suggests that this work will also be applicable to other cancer types.

## Supplementary information

supplementary material

## Data Availability

All bench-lab and anonymised clinical data and documentation can be provided on request. Raw or processed whole-exome sequencing (WES) data will be uploaded, following publication, to Dryad (https://datadryad.org/stash) or equivalent. Code script files can be provided upon request.
