# Supplementary material for: The DNA damage response in advanced ovarian cancer: functional analysis combined with machine learning identifies signatures that correlate with chemotherapy sensitivity and patient outcome
Source: Br J Cancer. 2023 Feb 21;128(9):1765–76. doi: 10.1038/s41416-023-02168-3 (PMC10133248; doi:10.1038/s41416-023-02168-3)
Supplement: Supplementary file 1 — supplementary material [file 41416_2023_2168_MOESM1_ESM.docx]

Supplementary Information

# Supplementary Methods

Supplementary methods referenced from manuscript methods section are detailed here.

## Explant characterisation antibodies

| Antibody target | Species | Company | Cat. number |
| --- | --- | --- | --- |
| Anti-Ca125 | Mouse monoclonal | Abcam | ab1107 |
| Anti-Pax8 | Mouse monoclonal | Abcam | ab92547 |
| Anti-Vimentin | Rabbit Monoclonal | Abcam | ab92547 |
| Anti-Pan Cytokeratin | FITC-conjugated Mouse Monoclonal | Merck | cbl234f |
| Anti-Mouse IgG | Goat, Alexa Fluor 546 | Invitrogen | A-11003 |
| Anti-Rabbit IgG | Goat, Alexa fluor 488 | Invitrogen | A-11008 |

## Rad 51 assay (HR) antibodies

| Antibody target | Species | Company | Cat. number |
| --- | --- | --- | --- |
| Anti-Mouse IgG | Goat, Alexa Fluor 546 | Invitrogen | A-11003 |
| Anti-Rabbit IgG | Goat, Alexa fluor 488 | Invitrogen | A-11008 |

## *In vitro* cell extract assay (NHEJ) (Optimisation & HGS-Optimisation cohorts)

#### Rationale

Non-homologous end joining was assessed by the ability of cell extracts to re-join linearized plasmid monomers into multimers. Two pGEM-3Zf(+)-derived plasmids were used for the assay (kindly donated by Dr Ann Kiltie (Oxford, UK)). The 1+3 plasmid generates a 3.2 kb plasmid with perfect 3’ overhang sequence match following BstXI digestion (CCACTAAG_GTGG and GGTG_ATTCCACC). Conversely the 1+4 plasmid generates a 3.2 kb plasmid 2bp mismatch 3’ overhang following BstXI digestion (CCACTAAG_GTGG and GGTG_AAACCACC).

#### Reagents

| **Buffers** | **Reagent** | **Concentration** |
| --- | --- | --- |
| **Hypotonic lysis buffer** | Tris HCl pH 8.0 | 10mM at pH 8.0  1mM  1mM |
|  | EDTA |  |
|  | DTT |  |
| **Protease Inhibitor** | PMSF | 0.17mg/ml  0.01U/ml  1ug/ml  1ug/ml  1ug/ml |
|  | Aprotinin |  |
|  | Pepstatin |  |
|  | Chymostatin |  |
|  | Leupeptin |  |
| **High Salt Buffer** | Tris HCl pH 7.5 | 50mM  1M  2mM  1mM |
|  | KCl |  |
|  | EDTA |  |
|  | DTT |  |
| **Dialysis Buffer** | Tris HCl pH 8.0 | 20mM  20% v/v glycerol  0.1M  0.5mM  1mM |
|  | Glycerol |  |
|  | KOAc |  |
|  | EDTA |  |
|  | DTT |  |
| **Deproteinization Mix** | Proteinase K | 10 mg/mL |
|  | SDS | 2.5% |
|  | EDTA | 50 mM |
|  | Tris-HCl pH 7.5 | 100 mM |
|  | H_2_O | To final volume |

#### Linearised DNA plasmid substrate preparation

10µg yields of each plasmid were digested in 50µl using BstXI in NEBuffer3 for 120 minutes at 37°C (New England Biolabs). Digested product was resolved on 1% agarose gels (60 minutes at 100 volts) and 3.2kb linearised bands were excised and purified using a QIAquick Gel Extraction Kit (Qiagen) as per manufacturer’s instructions. Purified linearised plasmids were adjusted to 5ng/µl following NanoDrop spectrophotometry and full BstXI digestion was confirmed by 35 cycle endpoint PCR with pGEM-3Zf(+) sequencing primer locations and 0.7% agarose gel electrophoresis compared to undigested plasmid control reactions.

#### Cell protein extract preparation

3x10^6^ cells were incubated in 500µl hypotonic buffer for 20 minutes on ice prior to homogenisation with a protease inhibitor cocktail (PMSF 170µg/ml, Aprotinin 1µg/ml, Pepstatin A 1µg/ml, Chymostatin 1µg/ml and Leupeptin 1µg/ml). Samples were incubated for 20 minutes on ice in the presence of 0.5 volume high salt buffer and centrifuged at 70,000rpm for 60minutes. Cell protein fractions were purified by recovering 90% of the supernatant underneath the lipid layer following centrifugation and samples dialyzed at low speed for 2hrs, at 4°C. Purified cell protein 10µl aliquots were stored at -80°C.

#### DNA End Joining Assay

20µl end-joining reactions were prepared that contained 200ng of either of the two plasmid DNA substrates, and 50µg of purified cell protein extract. Reactions were incubated in the presence of T4 DNA Ligase Reaction Buffer (New England Biolabs) for 2 hours at 37°C. Following the end-joining incubation samples were incubated with 80µg/ml RNase A for 10 minutes at 37°C (Thermo Fisher Scientific) and proteins were removed by incubation with 2mg/ml proteinase K and 0.5% w/v SDS for 10 minutes at 65°C. End-joined plasmid samples were resolved on 0.7% agarose gels and imaged on a GelDocIt system with Aida Image Analyser software.

#### Optional PCR confirmation of end-joined product

In addition to agarose gel visualisation of ligated plasmids (above), plasmid DNA was purified following the end joining assay using a QIAmp DNA blood mini kit (QIAGEN) to use as DNA template in PCR reactions that contained plasmid sequence primers. Thermocycler conditions were typical 35 cycle programs with a 55°C 30 second anneal step. PCR samples were resolved on a 2% agarose gel for 60 minutes at 100 volts and visualised.

## Host-cell reactivation assay (NHEJ) (Validation and HGS-Validation cohorts)

The Host-cell Reactivation system (Nagel *et al* ^1^) was further expanded to provide per-cell quantitative NHEJ pathway capacity monitoring of blunt end, 5’-3’ and 3’-5’ discontiguous de-phosphorylated mismatched overhang DNA double-strand breaks.

#### Host cell plasmid preparation

XL1-Blue supercompetent cells (200236; Agilent) were transformed with reconstituted pCMV6-AC-GFP plasmid (ps100010; Origene). Transformed cells were plated on LB agar (11508926; Invitrogen) containing 100µg/ml carbenicillin (C1389; Sigma) and incubated for 16 hours at 37°C. 20ml LB broth: 100µg/ml carbenicillin flasks were inoculated with transformed colonies and cultured for 8hrs at 37°C with 220rpm shaking. 1000ml step-up volumes were seeded from these and cultured under identical conditions for 16 hours in the presence of negative control flasks (non-transformed XL1-Blue cells, and no-cell media flasks). Plasmids were purified using endotoxin-free maxiprep kits and resuspended in endotoxin-free TE Buffer (12362; Qiagen). Purities and yields were assessed by UV spectrophotometry (NanoDrop ND-2000) and agarose gel analysis. ≈1000µg yields were obtained and plasmids were adjusted to 1000ng/ml concentrations.

EcoRI-HF (R3101S), SacI-HF (R3156S), KpnI-HF (R3142S), PmeI (R0560S), and Quick CIP (M0525) (New England Biolabs) were used with NEB CutSmart buffer and nuclease-free water to construct the three dephosphorylated double strand break (dsb) plasmid conformations provided below (condition 4 is uncut positive control plasmid).

| Condition | N-5’ | N-3’ | C-5’ | C-3’ | Blunt | Provides | Functioning NHEJ mediators likely required |
| --- | --- | --- | --- | --- | --- | --- | --- |
| 1 | EcoRI |  |  | KpnI |  | 5’ hang: 3’ hang | DNA-PKcs: Artemis endonuclease activity |
| 2 |  | SacI | EcoRI |  |  | 3’ hang: 5’ hang | Pol µ activity [Artemis likely required] |
| 3 |  |  |  |  | PmeI | Blunt | Ku–XRCC4–DNA ligase IV activity [Artemis not required] |


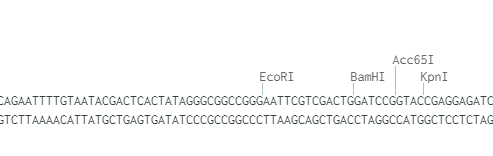


*15nt removed*

**Condition 1:** 5’ : 3’ double strand break

**Condition 2:** 3’ : 5’ double strand break


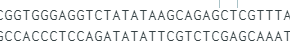

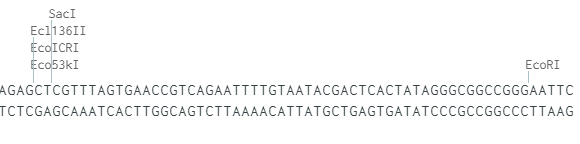


*52nt removed*

**Condition 3:** Blunt double strand break

*0nt removed*


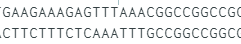

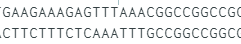


1µg 50µl restriction digest reactions were prepared with 20 units of each enzyme and 2n the suggested Quick CIP volume to obtain dephosphorylation. Reactions were incubated at 37°C for 4n the required time to achieve complete digestion and no more than 0.25n the minimum reported time prior to star activity, followed by heat-inactivation. Volumes were scaled to aliquots containing 10µg of plasmid as necessary in order to meet required yields.

2µg digested plasmid aliquots were resolved on 1% agarose gels alongside uncut reference plasmid and visualised by 1:1000 SYBR Safe (Thermo Fisher). dsb plasmid bands were gel extracted, purified, (QIAQuick Gel Extraction Kit 28704; Qiagen) and reconstituted in Endo-Free TE Buffer (12362; Qiagen). Plasmid purity and yields were assessed by agarose gel and spectrophotometry. From restriction digest preparation to digest purification (which incorporates gel-extraction), the inter-batch yield variation was 1% - 3% for the same plasmid conformation digests, and 1.7% - 7% yield variation between the three different plasmid conformations. Between-plasmid conformation purity ratio differences ranged between 0% - 0.6% (A260/280) and 1% - 3.5% (A260/230). These were indicative of robust and concordant dsb plasmid preparation and unlikely to contribute any bias towards final NHEJ quantitation analysis.

#### Explant plasmid transfection

Explant plasmid transfections were prepared with lipofectamine LTX Plus or 3000 (15338-100, 11668-027; Thermo Fisher) using Opti-Mem (11058021; Thermo Fisher) as per manufacturer’s guidelines. Conditions were determined empirically and used 52µl reactions with a final ratio of 2µl:0.5µg for LTX:DNA and 1.5µl:0.5µg for 3000:DNA. Replicate wells of 24 well plates were transfected with one of the four conditions (Uncut plasmid positive control, 5’-3’, 3’-5’, blunt-blunt) once explant cells were 85% confluent. Cultures were monitored at 24, 48, and 72 hours prior to analysis. Primary cells demonstrate documented challenges and a wide-range of transfection efficiencies via liposome-based transfection delivery methods. Any primary culture that exhibited suboptimal lipofectamine LTX transfection efficiencies were reanalysed using lipofectamine 3000 assays. Ultimately no explant was unamenable to optimal transfection assays.

#### Plasmid repair monitoring

Fluorescence-governed NHEJ pathway activity of each sample was assessed qualitatively by microscopy and quantitatively by flow cytometry. Cells were washed with PBS and visualised using a Zeiss Axio Observer Z1 microscope with Zeiss Zen 2.3 software. Parent cells confirmed absence of non-specific fluorescence whilst uncut plasmid provided positive transfection controls. For quantitative assessment the same samples were trypsinised, recovered, washed in PBS, and resuspended in 140µl 4°C sterile-filtered flow buffer (2% BS PBS). Samples remained on ice and cytometric fluorescence was determined on a BD Accuri C6 (Beckman Coulter). Cell-free control runs of media, PBS, flow buffer, deionised water, sheath fluid, decontamination fluid, cleaning fluid confirmed that zero non-specific events occurred at a frequency within two orders of magnitude below parent cell detection rate. The fluorescent detection extent of any non-specific events did not obfuscate negative control signals. Forward and side scatter gates were calibrated on parent cells and applied across corresponding explant NHEJ repair groups. Regions were calibrated on parent cells to determine their intrinsic fluorescence extent and regions were applied across corresponding NHEJ repair groups. Positive control samples confirmed explant transfection and permitted transfection efficiencies to be monitored. Fluorescence detection within dsb plasmid groups represents NHEJ pathway-governed processing and repair of DNA dsb to restore the GFP ORF and permit mRNA transcription and GFP protein expression. Extent of NHEJ pathway-governed repair was assessed with respect to both change in average fluorescence and change in number of cell events.

#### Average fluorescence intensity normalisation

Fluorescence change is concordant with traditional cell-free extract NHEJ assays and provides per-explant NHEJ pathway capacities. Average left-side region fluorescence intensities (defined as innate cell fluorescence as determined by parent cells) was subtracted from right-side region fluorescence intensity of dsb repair plasmids to provide between-explant normalised extent of fluorescence increase generated by NHEJ pathway-governed plasmid repair. Values were standardised to parent negative and transfection positive control conditions to provide percent-scaled capacities.

#### Cell event normalisation

Cell event monitoring enables per-cell resolution of intra-explant NHEJ repair capacity variance and thus enables detection of possible basal level NHEJ activity which would traditionally be obfuscated below aggregate-assay sensitivity thresholds. The ratio of event frequency of positive threshold cells to all sample cells was calculated to provide the proportion of cells within any sample that demonstrated repair activity. The event ratio of positive control samples represents the maximum achieved transfection efficiency per explant and was used to percentage-calibrate target condition group ratios.

#### NHEJ pathway competence score generation

An integrated NHEJ capacity scoring system was derived from equal-weighted contributions of cell event and fluorescent intensity standardised percent values. *Ordinal plasmid condition scores* were: Fully Defective (<5%); Basal Activity (≥5% & <15%); Low Activity (≥15% & <25%); Competent Activity (≥25% & < 35%); and High Activity (≥35%). *Plasmid condition scores* were combined to provide *Explant capacity scores* which measure overall explant NHEJ repair capacity. *Ordinal explant capacity scores* provided a 12-point scale (driven from the three input five-point scales).

## Single Cell Gel Electrophoresis Assay (BER & NER)

#### Comet slide preparation

Standard microscope glass slides were coated with 1% ultrapure agarose in ultrapure water (16500500, 10977035; Thermo Fisher) and maintained in the dark. Cells were pelleted for three minutes at 1000rcf 4°C, washed in sterile 4°C dPBS (D8537, Sigma), pelleted again and dPBS removed. Pellets were resuspended in 1% low melting point agarose (V2111; Promega) in dPBS, and samples were distributed across replicate comet slides and allowed to set. 0.5% LMP agarose was distributed over samples and allowed to set.

#### Lysis

Cells were lysed at 4°C for a minimum of 60 minutes protected from light. Pre-chilled 4°C lysis buffer was prepared fresh (2.5M NaCl, 200mM NaOH, 100mM Na_2_EDTA_2_H_2_0, 10mM Trisma base, 1% Triton X-100, 10% DMSO).

#### Electrophoresis

Slides were transferred into electrophoresis tanks and submerged in fresh 4°C electrophoresis solution (200mM NaOH; 1mM Na_2_EDTA•2H_2_0 (pH 10), in distilled water, confirmed pH >13.0) and incubated for 45 minutes at 4°C in the dark to permit DNA unwinding. Electrophoresis was conducted for 36 minutes at 1.5V/cm with a 2mm buffer height above the comet slide hyperplane.

#### Comet imaging and pathway competence score generation

Slides were visualised with a Zeiss Axio Observer Z1 with Zeiss Zen 2.3 software. The 172-megapixel tiled images were acquired using automated stage positioning to provide between 6,000 and 14,000 comets per each of the four explant conditions for BER or NER assays. The lowest 10 percentiles per each explant assay condition were removed to reduce uninformative values and the median percent DNA in tail per each condition was determined. DNA quantitative capacity metrics were calculated as *conditions* (4 – 1) – (3 – 1) – (2 – 1). Explant *condition* *three* percent DNA in tail values were summed with the median *condition* *two* equivalent value. These were compared with *condition* *four* to determine significant BER & NER pathway capacity changes (Kruskal Wallis, alpha = 0.05). Normalised survivorship class balance fold-change was calculated as comet frequency / *condition* *one* comet frequency. Extent of percent DNA in tail variance per condition was indicative of intra-explant heterogeneity.

## Whole Exome Sequencing (MMR)

Purified explant DNA (QIAamp DNA Mini, 51304; Qiagen) was assessed for purity and yield by NanoDrop ND-1000. SureSelect Human All Exon V6 (Agilent) exome libraries were prepared and samples were paired-end whole exome sequenced (WES) on a DNBSEQ™ NGS Platform (BGI Genomics, Hong Kong) with a per-sample 100x depth-of-coverage target. Trimmed and cleaned reads had a minimum Phred score of 34.4. These were aligned to human reference build Hg19 / GRCh37 using BWA ^2^ (99.954% average alignment) and variants called using SAMtools ^3^.

Explant MMR pathway capacity scores were generated based on frequency of observed mutations per each ontological impact level severity across the MMR gene cohort. MMR intra-gene and inter-gene mutation frequency aggregates were weighted by Impact level severities and scored ordinally as Competent; Functioning; Reduced; Perturbed; and Defective.

## Statistical analysis, capacity scoring, classification, modelling, validation, dimension reduction

#### Software

R ^4^ packages used in addition to Base R are provided below.

| Row | Package | Citation |
| --- | --- | --- |
| 1 | tidyverse | ^5^ |
| 2 | splitstackshape | ^6^ |
| 3 | gridGraphics | ^7^ |
| 4 | gridExtra | ^8^ |
| 5 | gplots | ^9^ |
| 6 | ggpubr | ^10^ |
| 7 | viridis | ^11^ |
| 8 | RColorBrewer | ^12^ |
| 9 | plotly | ^13^ |
| 10 | webshot | ^14^ |
| 11 | htmlwidgets | ^15^ |
| 12 | ic50 | ^16^ |
| 13 | drc | ^17^ |
| 14 | PharmacoGx | ^18^ |
| 15 | GRmetrics | ^19^ |
| 16 | plotROC | ^20^ |
| 17 | pROC | ^21^ |
| 18 | MASS | ^22^ |
| 19 | nnet | ^22^ |
| 20 | car | ^23^ |
| 21 | caret | ^24^ |
| 22 | e1071 | ^25^ |
| 23 | Mice | ^26^ |
| 24 | VIM | ^27^ |
| 25 | vcd | ^28^ |
| 26 | rstatix | ^29^ |
| 27 | Hmisc | ^30^ |
| 28 | heatmaply | ^31^ |
| 29 | psych | ^32^ |
| 30 | rmcorr | ^33^ |
| 31 | corrplot | ^34^ |
| 32 | rcompanion | ^35^ |
| 33 | survival | ^36^ |
| 34 | survminer | ^37^ |
| 35 | PerformanceAnalytics | ^38^ |

#### Platinum cytotoxicity scores

Explant response to carboplatin was dichotomised using a GR_50_ value of <48µM for sensitive classification and ≥48µM for resistant classification. This value was determined with reference to 1). Our distribution of explant GR_50_ clustering ranges; 2). The range of carboplatin and cisplatin explant and cell line cytotoxicity values in the literature with consideration to their differing efficacies; 3). The typically observed increase in GR_50_ µM value vs the equivalent IC_50_ value per explant due to growth-rate correction within the model; 4). Explant resistance reports in the literature in contrast to spheroid and organoids; 5). Known cell culture media sensitising components ^39-46^.

#### ROS scores

TBHP-negative DCFDA-treated replicate well Ex/Em 485/535 nm plate reader fluorescent values were subtracted from all TBHP treatment groups and ROS burden following increased TBHP treatment was converted to fold change events. Explants that were unable to recover the ROS assault to less than two-fold endogenous values were scored as perturbed.

#### Mitochondrial membrane health scores

Fluorescence values at Ex/Em 490/525 nm (loss of membrane potential) and 540/590 nm (active membrane potential) were used to classify mitochondrial membrane status. Cell-free background values were subtracted from all replicates and the intra-plate and inter-plate replicate mean and standard deviation assessed per parent or each drug concentration. Explant baseline mitochondrial membrane activity ratios and parent-normalised activity changes in response to the presence of drug were calculated and used to score mitochondrial status. Changes of less than parent-normalised 1.2FC across all drug treatments in conjunction with high parent cell mitochondrial membrane ratios were considered dysfunctional.

#### Patient summarised multi-explant culture scores

Patient-summarised multi-explant scores were generated by an algorithmic approach to accommodate potentially discordant explant capacities. This framework resolves discordance using weightings from significance values, quantitative capacity score, variance, class-balance, multi-tissue frequency, and extent of discordant states across all explants in question. BER and NER quantitative capacity score values were weighted by -Log KW p values and multi-explant average scores were generated. Binomial “has heterogeneity” and ordinal “consensus capacity” scores were introduced. MMR scores were averaged with an additional binomial “has heterogeneity” score and weighted for functioning explants. Averaged NHEJ ordinal scores were weighted for explant competence and binomial “has heterogeneity” and ordinal “consensus capacity” scores were introduced. Binomial metrics for the Rad51 (HR) assay can only by definition resolve to a summary value via odd explant numbers. Any equally weighted even number HR findings were ordinal-scored as heterogeneous as required and a binomial “consensus capacity” score introduced for any odd-number-explant collapse. Intra-tissue assay variance was retained with weightings for greatest input variance values. Class-balance values were retained with weightings for greatest survivorship bias. Cytotoxicity values were weighted for resistance and binomial “has heterogeneity” and ordinal consensus scores introduced. ROS and Mt membrane scores were weighted for dysfunction alongside “has heterogeneity” and binomial “consensus capacity” scores.

# Supplementary Data

## Supplementary Data section 1


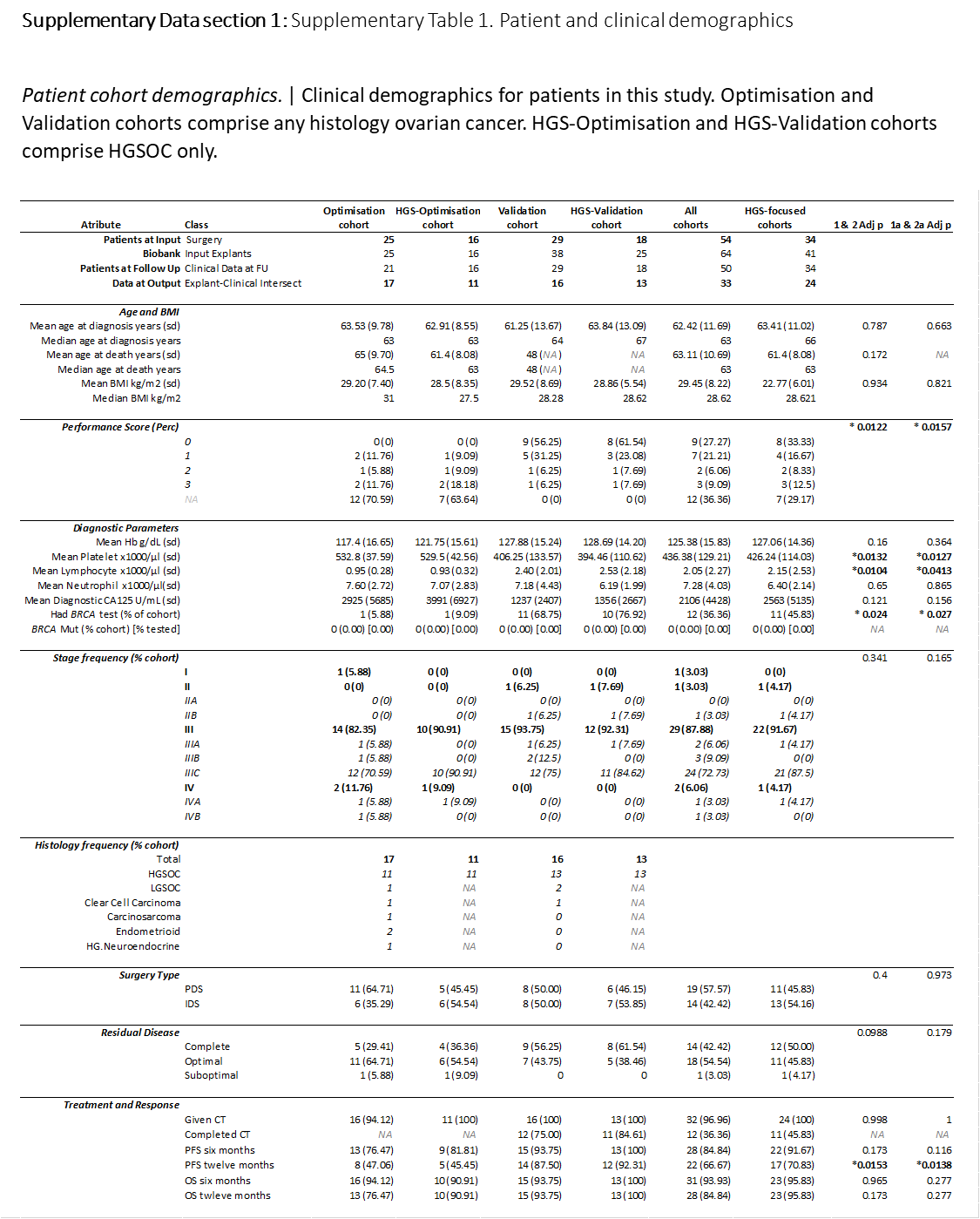


## Supplementary Data section 2


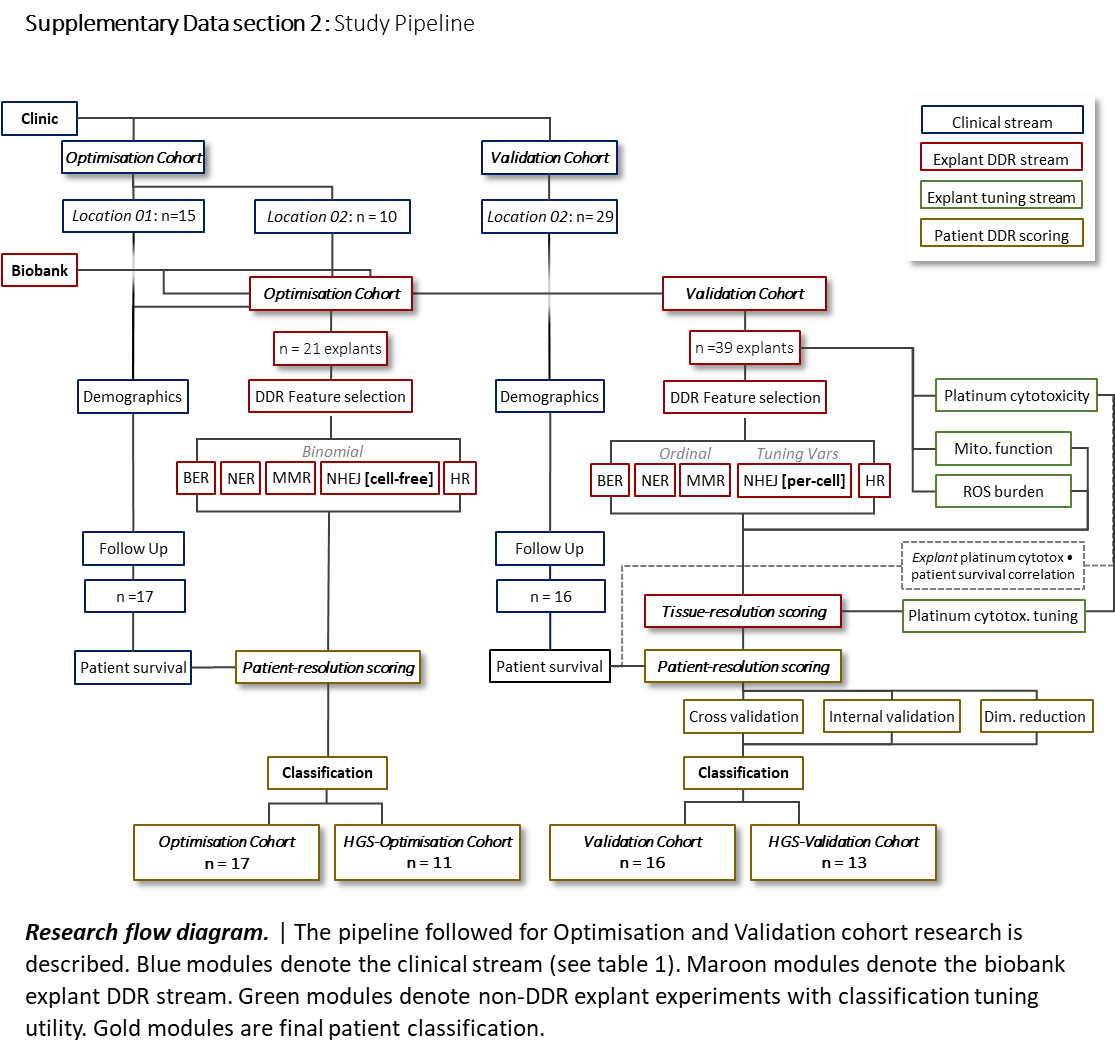


## Supplementary Data section 3


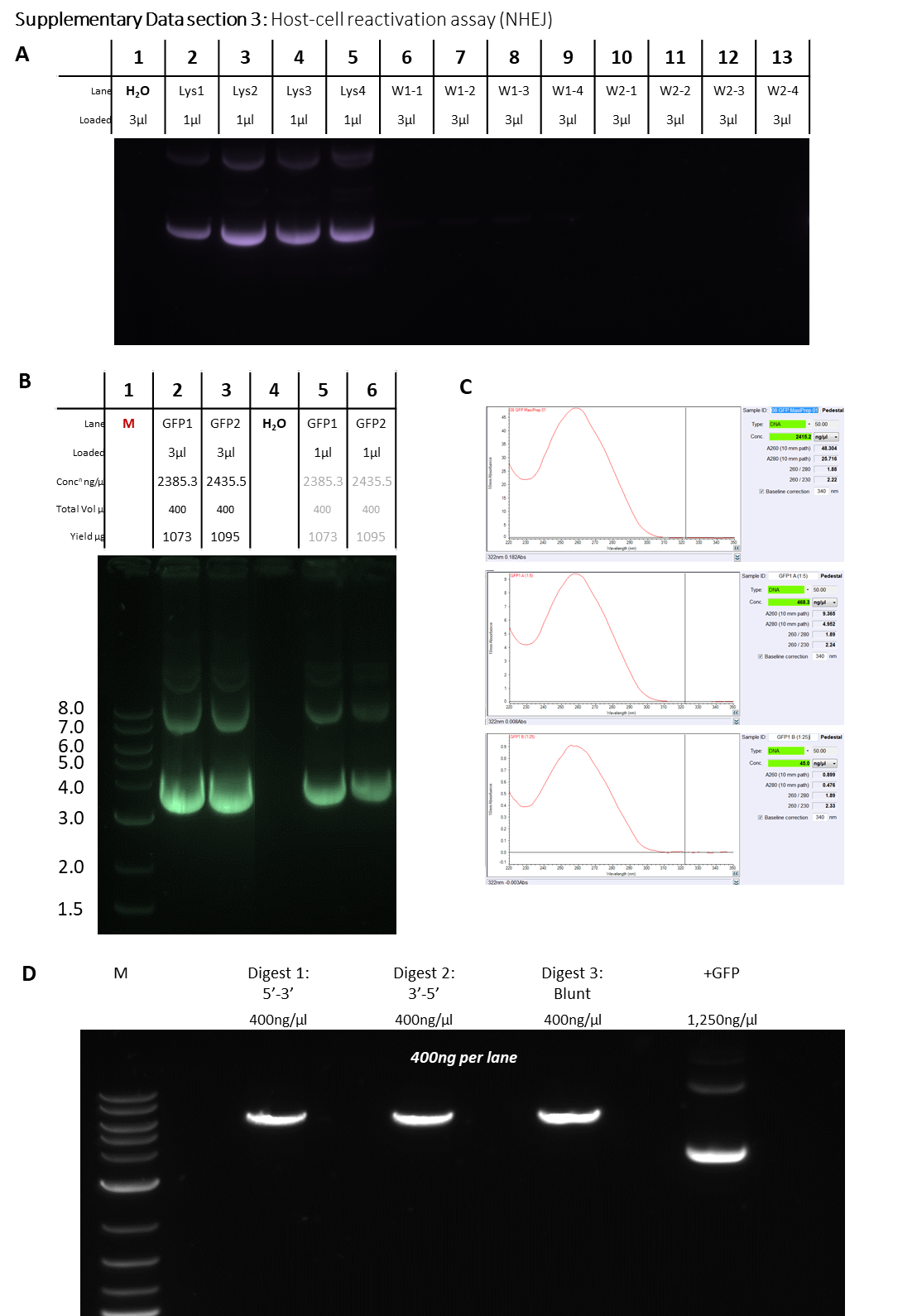

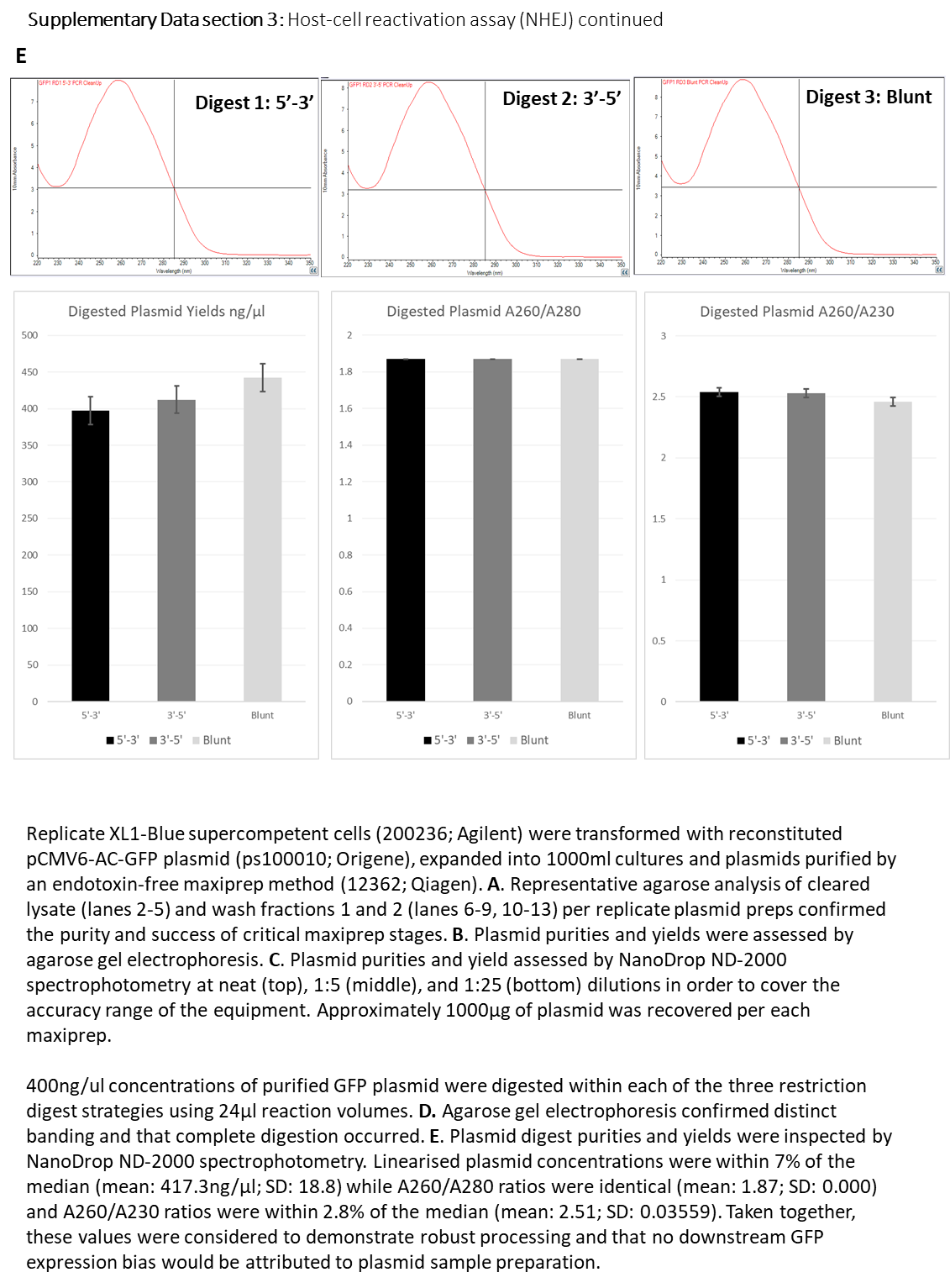


## Supplementary Data section 4


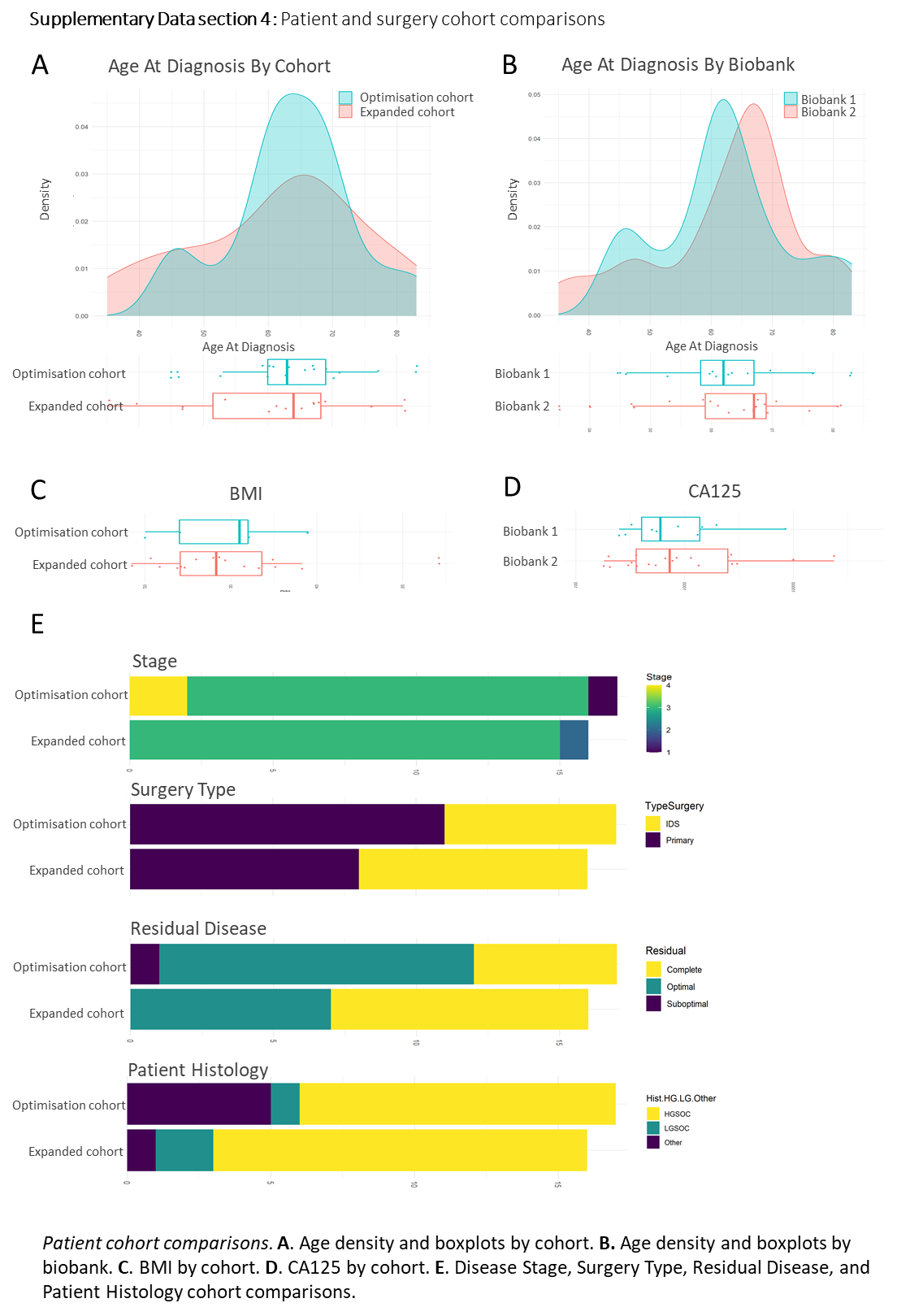


## Supplementary Data section 5


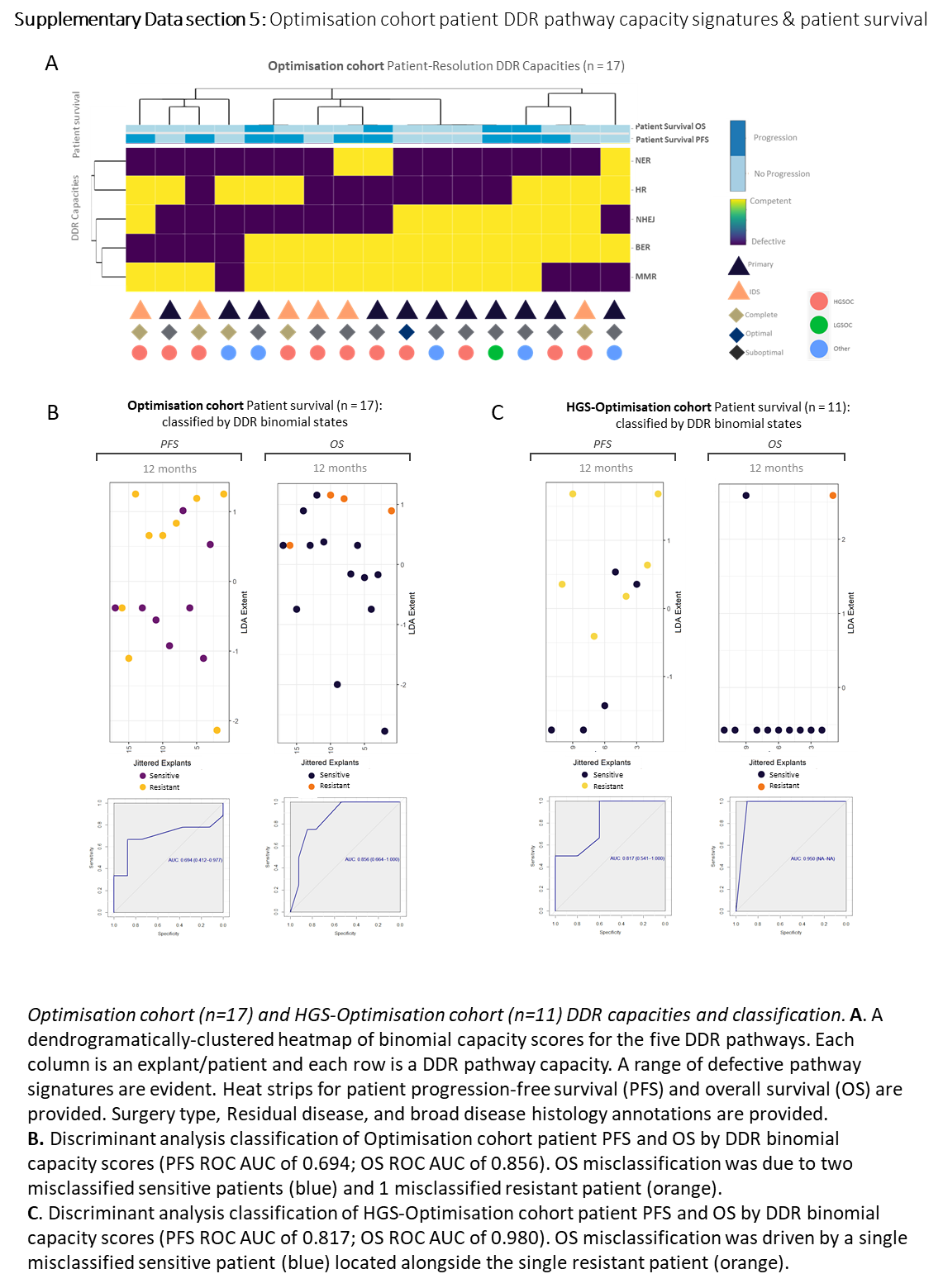


## Supplementary Data section 6


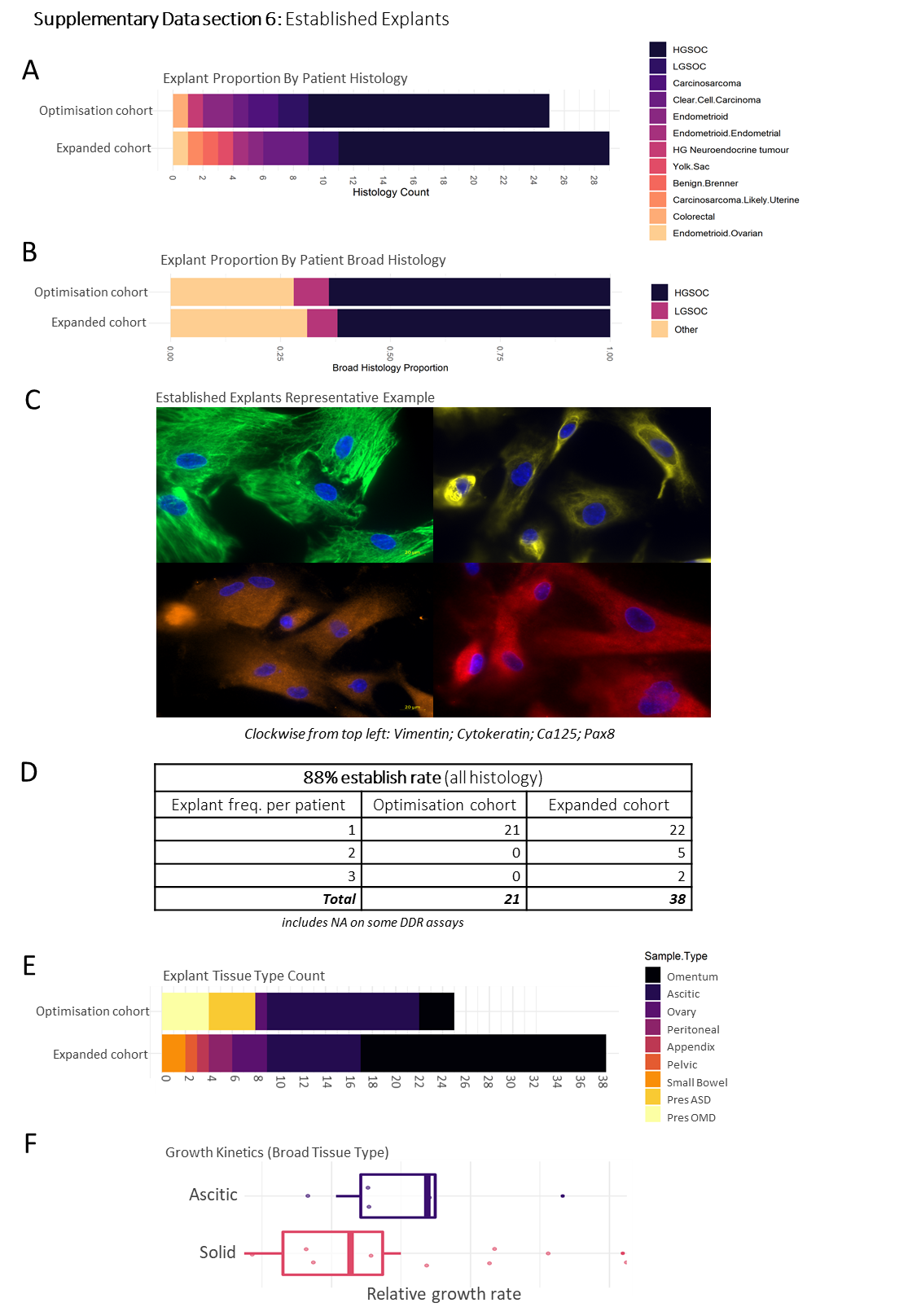


## Supplementary Data section 7


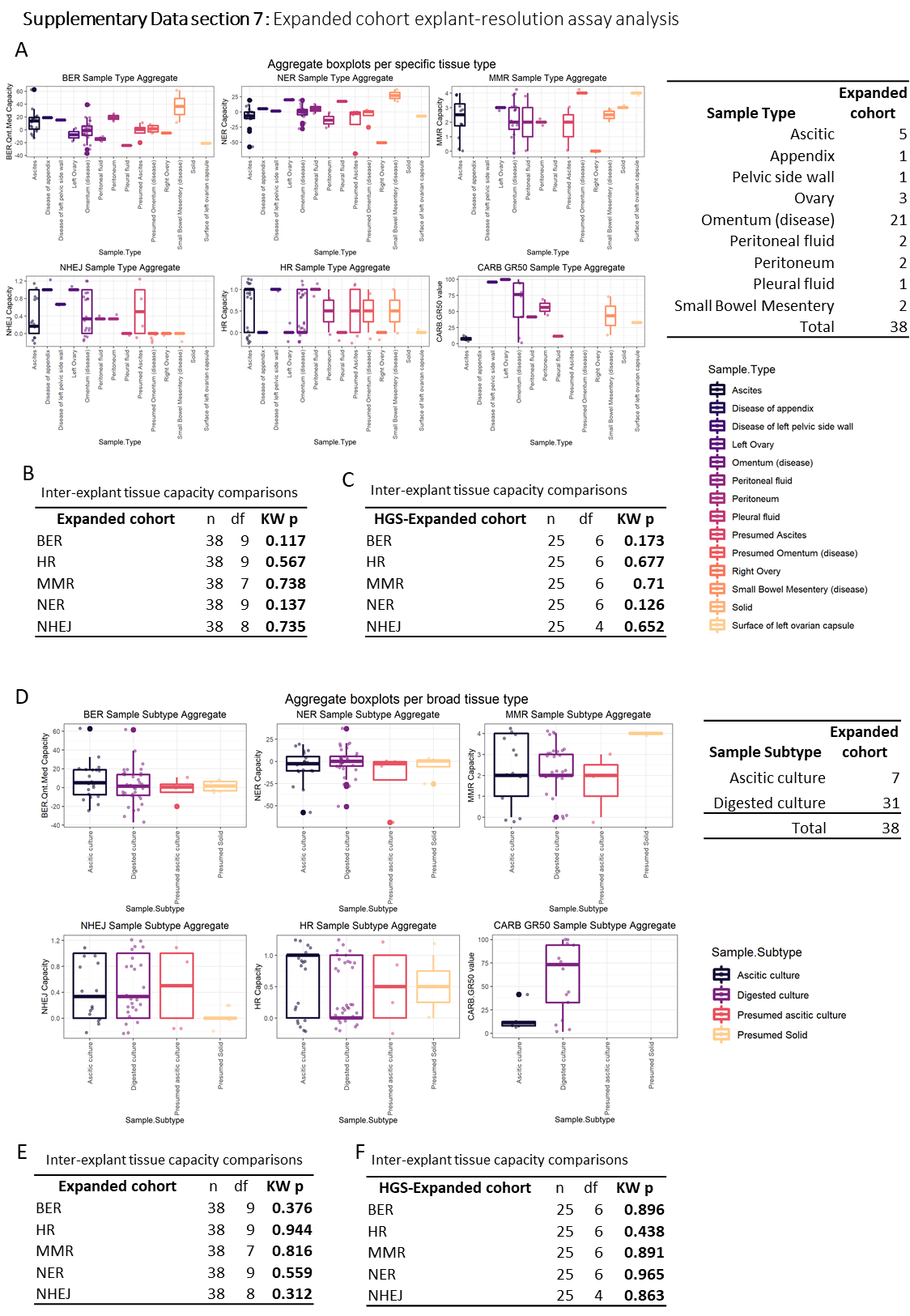

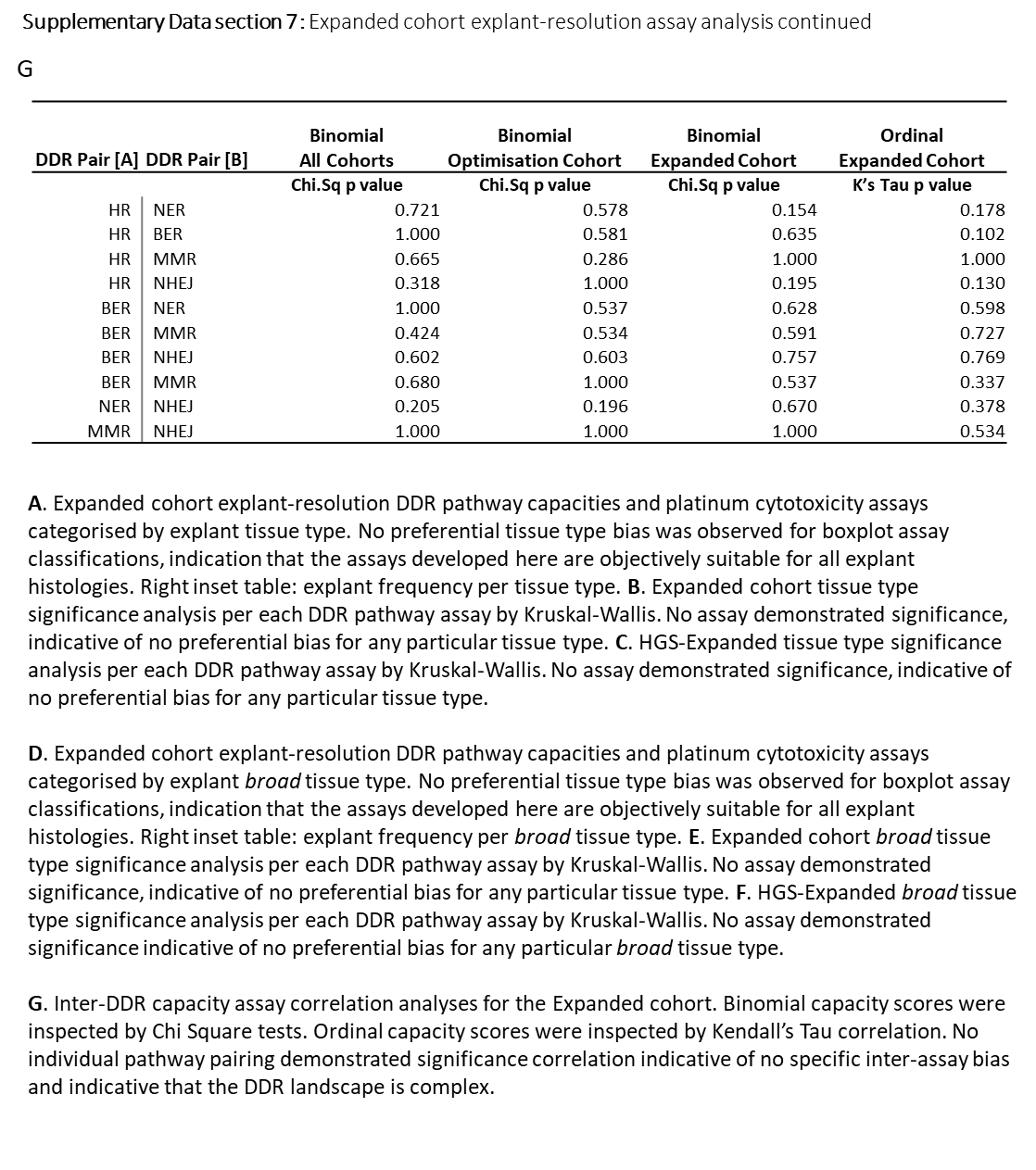


## Supplementary Data section 8


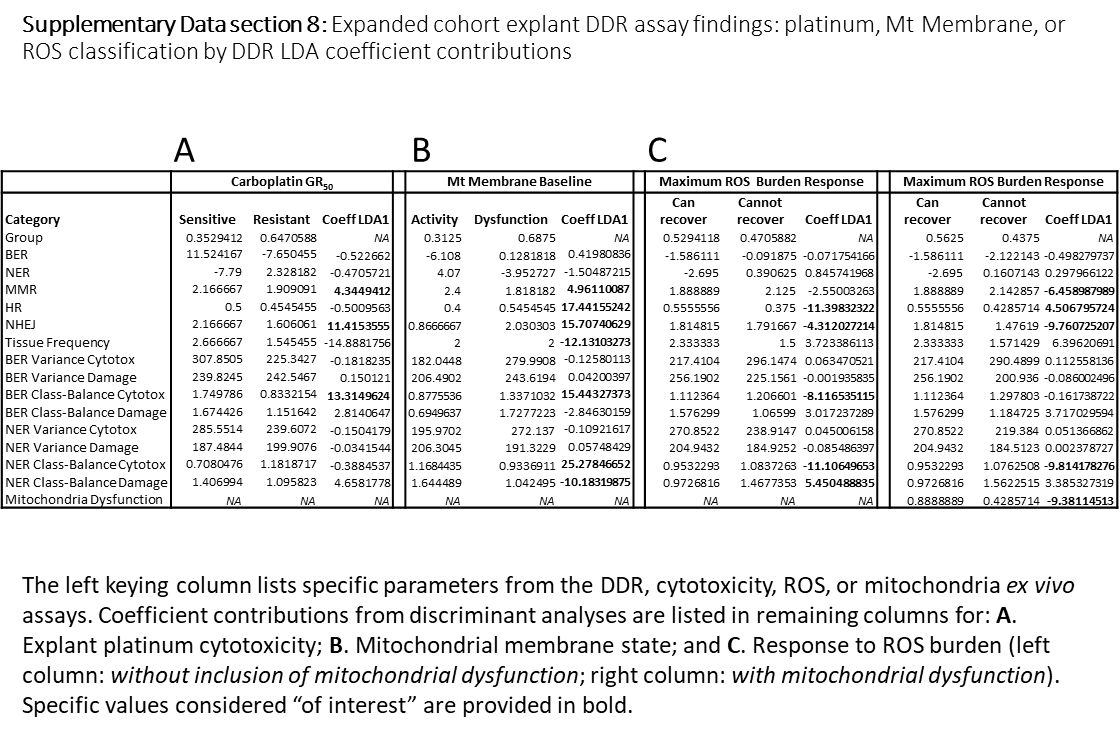


## Supplementary Data section 9


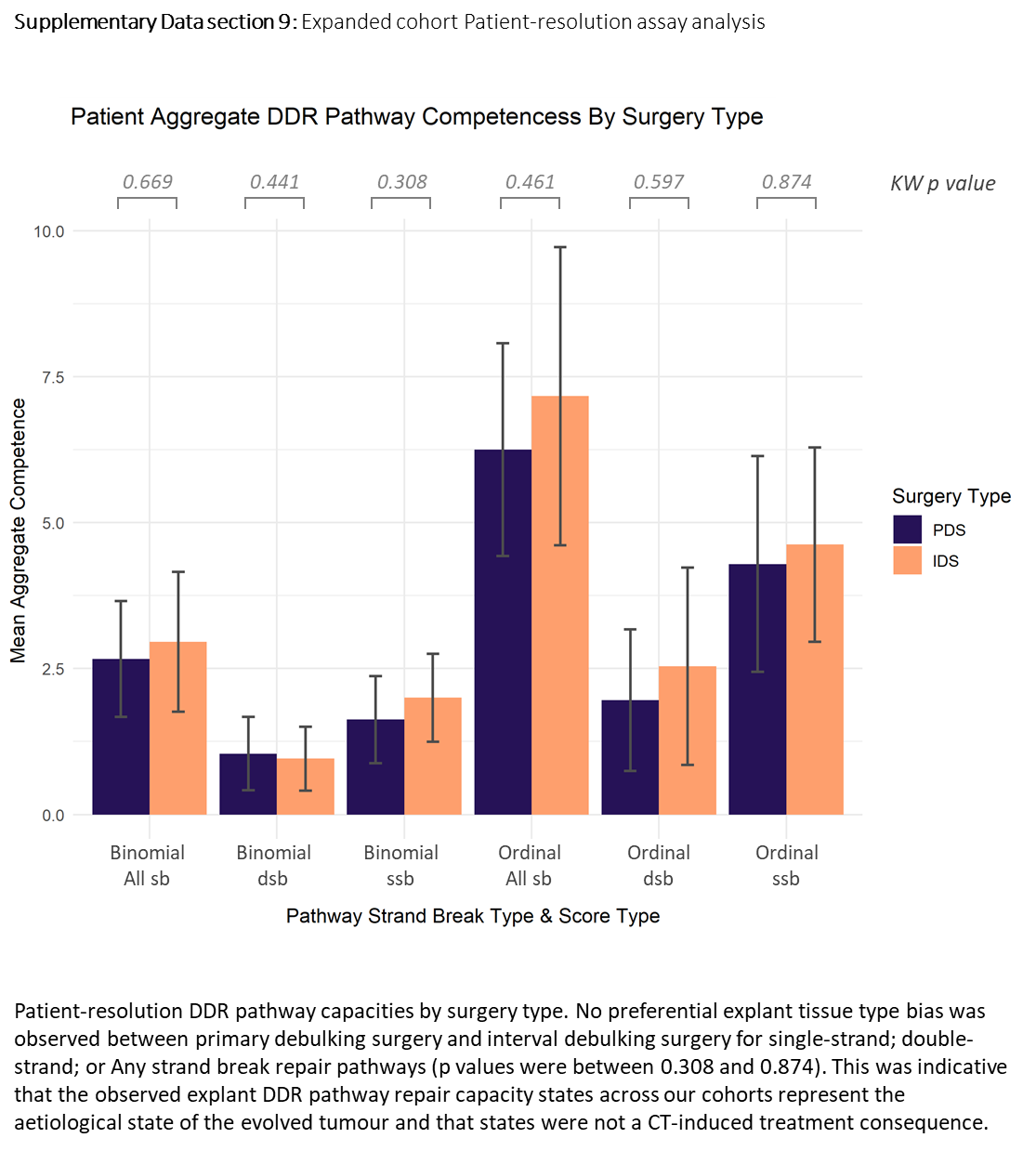


## Supplementary Data section 10

17 MOCs used for analysis. **Coloured by Resistant or Sensitive**. Vertical line is the 48µM threshold determined previously


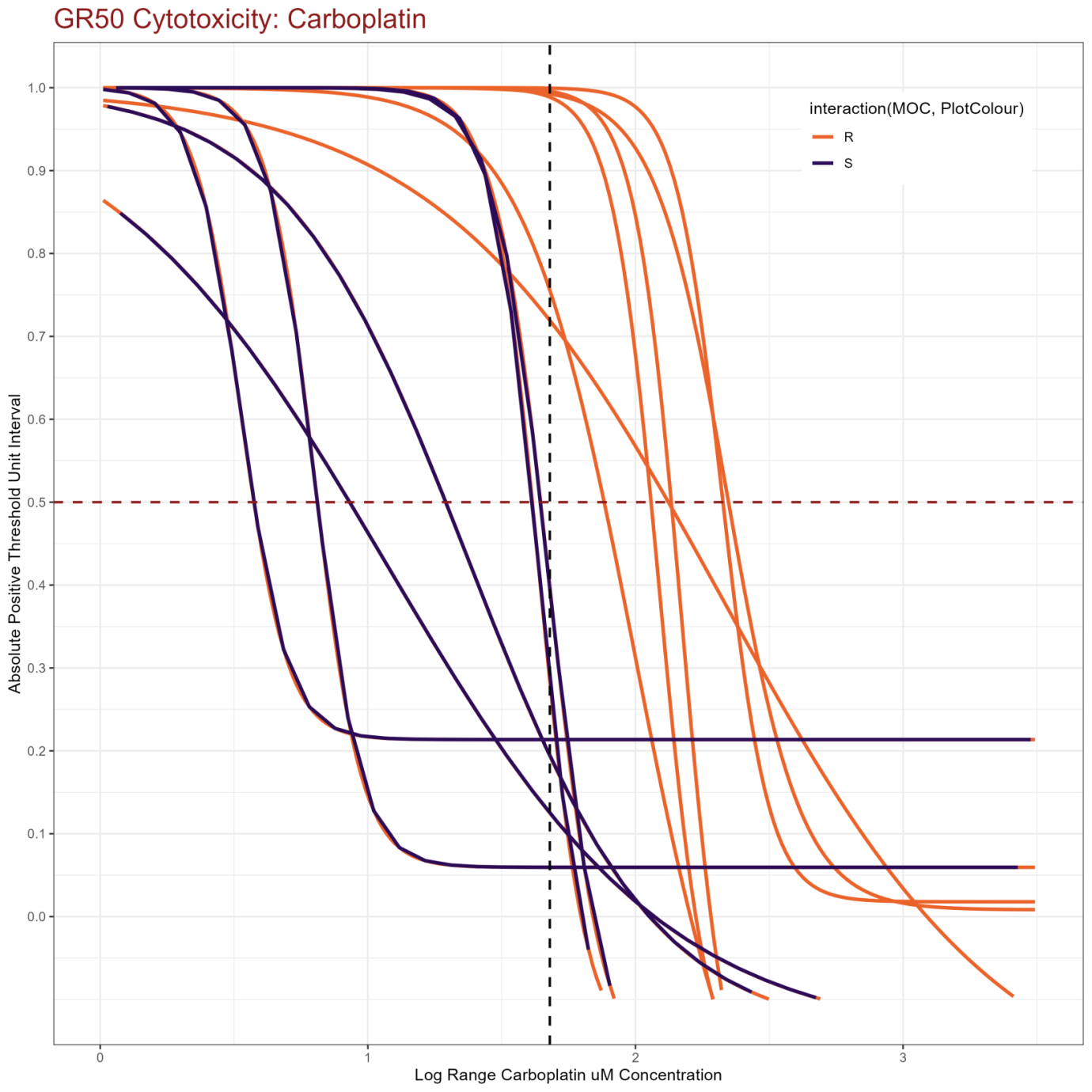


# References

1 Nagel, Z. D. *et al.* Multiplexed DNA repair assays for multiple lesions and multiple doses via transcription inhibition and transcriptional mutagenesis. *Proceedings of the National Academy of Sciences of the United States of America* **111**, E1823-E1832 (2014).

2 Li, H. & Durbin, R. Fast and accurate short read alignment with Burrows-Wheeler transform. *Bioinformatics* **25**, 1754-1760 (2009).

3 Li, H. *et al.* The Sequence Alignment/Map format and SAMtools. *Bioinformatics* **25**, 2078-2079 (2009).

4 R: A Language and Environment for Statistical Computing (2021).

5 Wickham, H. Welcome to the Tidyverse. *Journal of Open Source Software* **4**, 1686 (2019).

6 Mahto, A. splitstackshape: Stack and Reshape Datasets After Splitting Concatenated Values. (2019).

7 Murrell., P. The gridGraphics Package. *The R Journal}* **7**, 151--162 (2015).

8 Auguie, B. gridExtra: Miscellaneous Functions for "Grid" Graphics. (2017).

9 Warnes, G. R. *et al.* gplots: Various R Programming Tools for Plotting Data. (2020).

10 Kassambara, A. ggpubr: 'ggplot2' Based Publication Ready Plots. (2020).

11 Garnier, S. viridis: Default Color Maps from 'matplotlib'. (2018).

12 Neuwirth, E. RColorBrewer: ColorBrewer Palettes. (2014).

13 Sievert, C. *Interactive Web-Based Data Visualization with R, plotly, and shiny*. (Chapman and Hall/CRC, 2020).

14 Chang, W. webshot: Take Screenshots of Web Pages. (2019).

15 Vaidyanathan, R. *et al.* htmlwidgets: HTML Widgets for R. (2020).

16 Frommolt, P. ic50: Standardized high-throughput evaluation of cell-based compound screens. (2010).

17 Ritz, C., Baty, F., Streibig, J. C. & Gerhard, D. Dose-Response Analysis Using R. *PLOS ONE* **10**, e0146021 (2016).

18 Smirnov, P. *et al.* PharmacoGx: an R package for analysis of large pharmacogenomic datasets. *Bioinformatics* **32**, 1244-1246 (2015).

19 Hafner, M., Niepel, M., Chung, M. & Sorger, P. K. Growth rate inhibition metrics correct for confounders in measuring sensitivity to cancer drugs. *Nature Methods* **13**, 521-527 (2016).

20 Sachs, M. C. plotROC: A Tool for Plotting ROC Curves. *J Stat Softw* **79** (2017).

21 Robin, X. *et al.* pROC: an open-source package for R and S+ to analyze and compare ROC curves. *BMC Bioinformatics* **12**, 77 (2011).

22 Venables, W. N. & Ripley, B. D. Modern Applied Statistics with S. In: *Statistics and Computing* 10.1007/978-0-387-21706-2 (Springer New York, 2002).

23 Fox, J. & Weisberg, S. *An R Companion to Applied Regression*. (Sage: Thousand Oaks, CA, 2019).

24 Kuhn, M. Building Predictive Models in R Using the caret Package. *2008* **28**, 26 (2008).

25 David Meyer, E. D., Kurt Hornik, Andreas Weingessel and Friedrich Leisch. e1071: Misc Functions of the Department of Statistics, Probability Theory Group (Formerly: E1071), TU Wien. (2020).

26 van Buuren, S. & Groothuis-Oudshoorn, K. mice: Multivariate Imputation by Chained Equations in R. *2011* **45**, 67 (2011).

27 Kowarik, A. & Templ, M. Imputation with the R Package VIM. *2016* **74**, 16 (2016).

28 Meyer, D., Zeileis, A. & Hornik, K. The Strucplot Framework: Visualizing Multi-way Contingency Tables with vcd. *2006* **17**, 48 (2006).

29 Kassambara, A. rstatix: Pipe-Friendly Framework for Basic Statistical Tests. (2021).

30 Harrell.Jr, F. E. Hmisc: Harrell Miscellaneous. (2021).

31 Galili, T., O’Callaghan, A., Sidi, J. & Sievert, C. heatmaply: an R package for creating interactive cluster heatmaps for online publishing. *Bioinformatics* **34**, 1600-1602 (2017).

32 Revelle, W. psych: Procedures for Psychological, Psychometric, and Personality Research. (2020).

33 Bakdash, J. Z. & Marusich, L. R. Repeated Measures Correlation. *Frontiers in Psychology* **8** (2017).

34 Wei, T. & Simko, V. R package "corrplot": Visualization of a Correlation. (2017).

35 Mangiafico, S. rcompanion: Functions to Support Extension Education Program Evaluation. (2021).

36 Therneau, T. A Package for Survival Analysis in R. (2020).

37 Kassambara, A., Kosinski, M. & Biecek, P. survminer: Drawing Survival Curves using 'ggplot2'. (2020).

38 Peterson, B. G. & Carl, P. PerformanceAnalytics: Econometric Tools for Performance and Risk Analysis. (2020).

39 Bicaku, E. *et al.* In vitro analysis of ovarian cancer response to cisplatin, carboplatin, and paclitaxel identifies common pathways that are also associated with overall patient survival. *British Journal of Cancer* **106**, 1967-1975 (2012).

40 Fang, F. *et al.* Genomic and Epigenomic Signatures in Ovarian Cancer Associated with Resensitization to Platinum Drugs. *Cancer Research* **78**, 631-644 (2018).

41 Fotopoulou, C. Limitations to the use of carboplatin-based therapy in advanced ovarian cancer. *EJC Suppl* **12**, 13-16 (2014).

42 Haley, J. *et al.* Functional characterization of a panel of high-grade serous ovarian cancer cell lines as representative experimental models of the disease. *Oncotarget* **7**, 32810-32820 (2016).

43 Patra, B. *et al.* Carboplatin sensitivity in epithelial ovarian cancer cell lines: The impact of model systems. *PloS one* **15**, e0244549-e0244549 (2020).

44 Rantanen, V., Grénman, S., Kulmala, J. & Grénman, R. Comparative evaluation of cisplatin and carboplatin sensitivity in endometrial adenocarcinoma cell lines. *British journal of cancer* **69**, 482-486 (1994).

45 Sonego, M. *et al.* Common biological phenotypes characterize the acquisition of platinum-resistance in epithelial ovarian cancer cells. *Sci Rep* **7**, 7104 (2017).

46 Su, W. C., Chang, S. L., Chen, T. Y., Chen, J. S. & Tsao, C. J. Comparison of in vitro growth-inhibitory activity of carboplatin and cisplatin on leukemic cells and hematopoietic progenitors: the myelosuppressive activity of carboplatin may be greater than its antileukemic effect. *Jpn J Clin Oncol* **30**, 562-567 (2000).
